# Optimising Analysis Choices for Multivariate Decoding: Creating Pseudotrials Using Trial Averaging and Resampling

**DOI:** 10.1111/ejn.70601

**Published:** 2026-07-19

**Authors:** Catriona L. Scrivener, Tijl Grootswagers, Alexandra Woolgar

**Affiliations:** ^1^ MRC Cognition and Brain Sciences Unit University of Cambridge Cambridge UK; ^2^ School of Philosophy, Psychology and Language Sciences University of Edinburgh Edinburgh UK; ^3^ The MARCS Institute for Brain, Behaviour and Development Western Sydney University Sydney New South Wales Australia; ^4^ School of Computer, Data and Mathematical Sciences Western Sydney University Sydney New South Wales Australia; ^5^ Department of Psychology University of Cambridge Cambridge UK

**Keywords:** decoding, multivariate pattern analysis, pseudotrials

## Abstract

Multivariate pattern analysis (MVPA) is a popular technique that can distinguish between condition‐specific patterns of activation. Applied to neuroimaging data, MVPA decoding for inference uses above chance decoding to identify statistically robust condition‐specific information in neuroimaging data, which may be missed by univariate methods. However, several analysis choices influence decoding results, and the combined effects of these choices have not been fully evaluated. In particular, an increasingly popular approach is to average data from several trials together before training an MVPA classifier, but the decision about how much averaging to do is arbitrary and the effect of varying this parameter has not been documented. Here, we systematically assessed the influence of trial averaging and resampling on decoding accuracy and subsequent statistical outcome on data simulated using two different toolboxes (CoSMoMVPA and SEREEGA). Although the optimal parameters varied with the classifier and cross‐validation approach used, we found that modest trial averaging using up to 5%–10% of the total number of trials per condition improved decoding accuracy and associated *t*‐statistics. In addition, a small amount of resampling could improve *t*‐statistics and classification performance, but was not always necessary. We provide code to allow researchers to optimise these analysis choices for the parameters of their data.

## Graphical abstract

We used simulated data to assess the influence of common analysis decisions, especially trial averaging and resampling, on decoding accuracy for multivariate pattern analysis (MVPA). Optimal parameters varied with the classifier and cross‐validation approach, but in all cases modest trial averaging using up to 5%–10% of the total number of trials per condition improved decoding accuracy and associated *t*‐statistics. A small amount of trial resampling improved *t*‐statistics and classification performance in some cases, but was not always necessary.

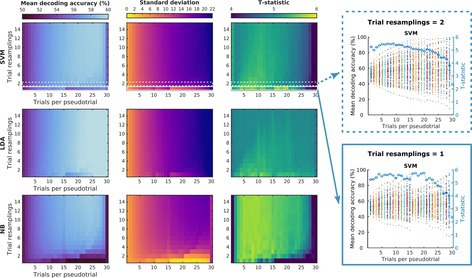

**Abbreviations**
LDA: linear discrimination analysis
MVPA: multivariate pattern analysis
NB: Naïve Bayes
SVM: support vector machine

## 1 Introduction

The last decade has seen an explosion in the popularity of multivariate pattern analysis (MVPA) for neuroimaging data. By identifying condition‐specific patterns of activation, MVPA can reveal the evolution of information processing over time and/or space and is sensitive to information missed by univariate methods (e.g., Grootswagers et al. 2017; Pereira et al. 2009; Haynes and Rees 2006). Typically, cognitive neuroscience experiments employ MVPA techniques, such as linear classification (‘decoding’), to make inferences about the type of information decodable from neuroimaging data and to characterise these ‘neural representations’ in terms of when and where they can be decoded, whether they generalise between conditions and if they change with experimental manipulations. Decoding for inference typically compares decoding metrics (e.g., classification accuracy) between conditions or to chance, drawing inference about neural processing from statistically reliable condition‐specific information in neuroimaging data.

There are many ways to run a decoding for inference analysis, and multiple decisions are likely to influence decoding success. One analysis option is the creation of ‘pseudotrials,’ or ‘supertrials,’ by averaging data from subsets of trials together before performing MVPA. This averaging approach assumes that each repeat of individual stimuli will be similar and that averaging would not remove any meaningful variation across repeats. This is conceptually similar to more traditional EEG analysis, such as ERPs (event‐related potentials), where all of the trials for each condition are averaged together before analysis. Previous results demonstrate that pseudotrial averaging can lead to an increase in classification accuracy, compared to single‐trial decoding (e.g., Adam et al. 2020; Tuckute et al. 2019; Hebart and Baker 2018; Grootswagers et al. 2017; Isik et al. 2014). However, too much averaging can be detrimental, as increasing the number of trials per pseudotrial can also increase the between‐subject variance in decoding accuracy, which in turn affects the statistical outcome.

A second decision is the cross‐validation procedure used to evaluate the generalisation of classification across subsets of the data (Bishop and Nasrabadi 2006). In a leave‐one‐trial‐out procedure, the number of cross‐validation folds is equal to the number of trials available per exemplar. At each fold, the classifier is trained on all but one of the trials, which is then used to test the classification. A leave‐one‐pseudotrial‐out method uses the same logic, but is based on averaged subsets of the original trials. Another option is to divide the data into larger chunks or blocks across which to train and test the classifier. For example, in a study with 90 trials per exemplar, 10‐fold cross‐validation would split the data into nine sets of 10 trials to use iteratively for training and testing. This means that at each iteration, the classifier is trained on fewer data points than for leave‐one‐trial‐out, but previous work has shown that this may provide more stable estimates with lower variance across decoding accuracies (Varoquaux et al. 2017). When combined with trial averaging, pseudotrials can be created either by averaging all of the trials within a chunk or by grouping them into smaller subsets of trials. Therefore, at least two interacting parameters seem likely to affect results: the number of trials averaged together before classification and the number of folds into which the data are split for cross‐validation.

Thus far the choice of these parameters has been largely arbitrary, resulting in a wide variety of trial averaging and cross‐validation approaches in the literature. To name a few examples, in Bae and Luck (2018) the available trials per condition were randomly divided into three chunks before being averaged and decoded using a 3‐fold cross‐validation. In Isik et al. (2014), groups of 10 trials were averaged before using a 5‐fold cross‐validation, and in Duncan et al. (2023), groups of three trials were averaged before using a 10‐fold cross‐validation. Goddard et al. (2022) averaged over 16 trials before using an 8‐fold cross‐validation, and Petit et al. (2023) used the median of five trials and a leave‐one‐pseudotrial‐out cross‐validation with 22 folds. The authors here used the median, rather than the mean, to reduce the impact of noisy trials. They opted to keep these trials rather than remove them to keep their paired design balanced. Given the variation across experiments, it can be difficult to make decoding analysis decisions regarding a new set of data.

In an evaluation of different decoding approaches, Grootswagers et al. (2017) compared decoding accuracies when averaging together 4, 8, 16 and 32 trials (equivalent to creating 8, 4, 2 and 1 pseudotrials) from an experiment of 32 trials per condition (64 total trials). A leave‐one‐pseudotrial‐out cross‐validation scheme was used, meaning that the number of cross‐validation folds was determined by the number of pseudotrials. All averaging procedures increased decoding accuracies compared to no trial averaging, but the least amount of averaging (4 trials, 12.5%) provided the best trade‐off between signal‐to‐noise and number of pseudotrials. In addition, they found similar decoding accuracies for 10‐fold and leave‐one‐trial‐out cross‐validation methods. They reported lower decoding accuracies using a 2‐fold cross‐validation procedure, though others have argued that this approach may be associated with higher statistical power overall (Valente et al. 2021). Zhang et al. (2025) tested a similar approach evaluating the number of trials per average and the number of cross‐validation folds, using a range of open EEG datasets. As in Grootswagers et al. (2017), all trials within each fold were averaged to create a single pseudotrial per fold. Across seven commonly used cognitive paradigms, they found optimal decoding outcomes using approximately 10–20 trials per average and between three and five cross‐validation folds.

In a different implementation, Adam et al. (2020) compared trial averaging results using a 3‐fold classification that was independent from the number of pseudotrials created. They averaged groups of trials within each fold, ranging from 5 to 25 and found a significant increase in the average decoding accuracy with more averaging. However, given the associated increase in between‐subject variance, they concluded that the average of 10 trials (this corresponded to between 2.5% and 10% of the available trials across datasets of different sizes) was optimal for the number of trials available. Thus, while there is clearly a trade‐off between providing a classifier with fewer less noisy trials (more averaging) or more noisy trials (less averaging), it is not yet clear how to optimise this decision. In addition, it is unclear whether and how the optimal amount of averaging depends on other factors such as number of trials, choice of classifier, effect size, etc.

The aim of the current work was to inform future decoding studies by systematically assessing the influence of a range of parameters on decoding accuracy and subsequent statistical outcome, using data simulated in CoSMoMVPA (Oosterhof et al. 2016) and SEREEGA (Krol et al. 2018). We varied several parameters across simulations, including the number of trials per pseudotrial, cross‐validation procedure, number of trials per condition and the size of the underlying effect, and assessed the data for three different linear classifiers. By providing a summary of how these parameters influence decoding outcomes, we aim to help readers make informed choices before analysis so that they do not need to test many different options. Decoding results varied with the classifier used, the cross‐validation approach and the number of trials averaged to create each pseudotrial. In addition, we evaluated the influence of random sampling with replacement (‘resampling’) on decoding accuracy, which has not yet been comprehensively assessed. We hypothesised that this would be beneficial when creating pseudotrials as it increases the number of samples available for classification, which would normally be diminished by trial averaging. Although the optimal parameters varied with classifier and cross‐validation approach, we found that using roughly 5%–10% of the total number of trials per condition was optimal for creating pseudotrials. In addition, a resampling value of 2 could improve *t*‐statistics and reduce the impact of the number of trials per pseudotrial on classification performance.

## 2 Methods

### 2.1 Simulation 1

First, we used CoSMoMVPA (Oosterhof et al. 2016) to simulate multiple datasets, each with two experimental conditions, and examined the influence of (a) the number of trials used per pseudotrial, (b) the number of times each trial was resampled, (c) the simulated class distance and (d) the choice of classifier. We chose three popular classifiers that are implemented in CoSMoMVPA: linear support vector machine (libSVM), linear discriminant analysis (LDA) and Gaussian Naïve Bayes. For experiment 1, we simulated data with 700 features (reflecting channels/voxels) from 100 ‘subjects’ with two conditions and 90 trials per condition. To check whether the results were dependent on the specific number of trials per condition, we also simulated a second experiment with only 45 trials per condition. We manipulated the multivariate class distance using built‐in CoSMoMVPA functions: Individual values were drawn from a normal distribution (standard deviation = 1) with the amount of separation between class‐means for each feature defined as class‐mean = class_distance/sqrt (log(700*ntrials)) with *ntrials* either 90 or 45 (for the 2 experiments), and used three values for *class_distance* (0, 0.1 and 0.2). We refer to a simulated class distance of 0 as ‘no effect,’ 0.1 as a ‘small effect’ and 0.2 as a ‘large effect.’ Results for a smaller number of participants (50 participants, Supplementary Material S4, Figure S6) and fewer features (6 features, Supplementary Material S5, Figure S7) are reported in the Supplementary. Simulation scripts are available on the Open Science Framework, https://osf.io/hjf75/.

**FIGURE 1:**
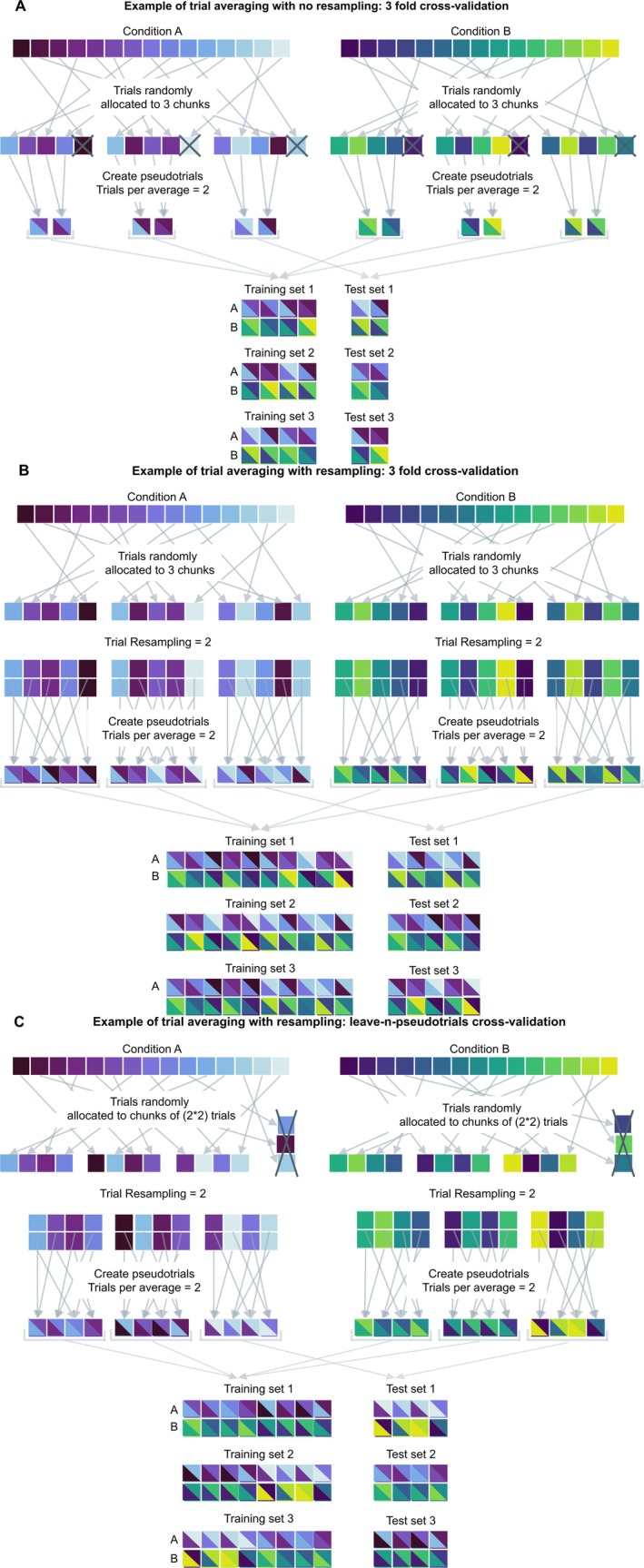
(A) Example of a 3‐fold cross‐validation decoding scheme with averaging but no resampling. In this example we have 15 trials for each condition (A and B). First, the trials for each condition are randomly allocated to three chunks, with five different trials in each chunk. If the number of trials did not divide by 3, a random selection of the remaining trials would be discarded. Next, we create pseudotrials using two trials per average (with no resampling), so we can create two unique pseudotrials per chunk, discarding the remaining trial in each chunk (indicated by the dark grey crosses). This creates six pseudotrials per condition. For each fold of the 3‐fold cross validation scheme, we train on the pseudotrials from two chunks (4 pseudotrials per condition) and test on the two pseudotrials in the remaining chunk. This is repeated until all of the chunks have been used once as the test set. We allocated the same number of trials to each chunk, and individual trials could only be allocated to one chunk (i.e., the trials allocated to each chunk are different). This ensures that the same trial can never be in more than one chunk and can never contribute to both a training and a test pseudotrial, ensuring that train and test sets are fully independent. (B) Example of a 3‐fold cross‐validation decoding scheme with both averaging and resampling. In this example we have 15 trials for each condition (A and B). First, the trials for each condition are randomly allocated to three chunks, with five different trials in each chunk. Trials are then resampled within each chunk (here: we use each trial twice). From this pool of resampled trials, we create pseudotrials within each chunk, using two trials per average, to create five unique pseudotrials per chunk. This yields 15 pseudotrials overall per condition. For each fold of the 3‐fold cross validation scheme, we train on the pseudotrials from two of the chunks (10 pseudotrials per condition) and test on the five pseudotrials in the remaining chunk. This is repeated until all of the chunks have been used once as the test set. As before, the same trial can never contribute to pseudotrials in multiple chunks, so the train and test sets are fully independent. (C) Example of a leave‐n‐pseudotrials out cross‐validation decoding scheme with both averaging and resampling. In this example we again have 15 trials for each condition (A and B). We want to use two trials per average and have a trial resampling of 2. To achieve this, we allocate four different randomly selected trials to each chunk (chosen as 2 trials per average × 2 trial resamplings = 4 trials per chunk). As we have 15 trials per condition, this means that we can create three chunks and use a 3‐fold cross‐validation scheme (15/4 = 3.75). A random selection of three trials are left out for each condition (indicated by the dark grey crosses). We then resample the trials within chunk (for a total of 8 trials per chunk). Within each chunk, we create pseudotrials using two trials per average, yielding four unique pseudotrials per chunk. This creates 12 pseudotrials per condition. For each fold of the n‐fold validation scheme (here: 3‐fold), we train on all but one chunk (here 2 chunks, for a total of 8 pseudotrials per condition) and test on the remaining chunk (here with 4 pseudotrials per condition). Because pseudotrials are created within each chunk, holding out entire chunks (rather than single pseudotrials) for the testing set ensures that the train and test sets are fully independent.

We ran both a ‘chunking’ and a ‘leave‐n‐pseudotrials‐out’ cross‐validation procedure as both approaches are commonly used. For the ‘chunking’ procedure, we created pseudotrials separately within each of three chunks of trials and used a 3‐fold cross‐validation method (additional results for a 10‐fold cross‐validation procedure can be found in the Supplementary Material S6, Figure S8). Note that in all cases, we allocate the same number of trials to each chunk. Pseudotrials were always created within chunk, and pseudotrials within a chunk were always kept together in the train/test scheme. Therefore, the same trial could never contribute to both a training and testing set, ensuring their independence (Grootswagers et al. 2017; Kriegeskorte et al. 2009). See Figure 1A for an example of the 3‐fold cross‐validation procedure with trial averaging, and Figure 1B for an example with both averaging and resampling.

For the ‘leave‐n‐pseudotrials‐out’ method, the number of cross‐validation folds was determined by both the number of trials per pseudotrial and the amount of trial resampling (number of folds = total experiment trial number before resampling/(trials per average*trial resampling), with a fixed minimum of 2 folds). In the simplest case with no trial resampling, the number of trials per fold is determined by the number of trials per average and can be thought of as a ‘leave‐one‐pseudotrial‐out’ approach. For example, if we have 90 trials and use 10 trials per average, then we are able to make nine pseudotrials and have nine cross‐validation folds (with 1 pseudotrial per chunk). If we instead use five trials per average, then we can create 18 pseudotrials and have 18 cross‐validation folds.

In the case of trial resampling, we allocate more trials to each fold to accommodate the resampling. In this case, the number of pseudotrials per fold (n) is determined by the resampling value, and we therefore use the term ‘leave‐n‐pseudotrials‐out’ to describe this method. It is essential that resampling is done strictly within fold, to ensure independence of training and testing sets. In addition, to avoid resampling the same trial twice in any single pseudotrial, we chose to allocate n trials per average * resampling trials to each fold. For example, if we want to use 10 trials per average with a resampling of 2, we now need to allocate 20 trials to each fold. If we simply allocated 10 trials, resampling of 2 would result in two identical averages of the 10 trials (as we do not allocate more than one of each trial to the same pseudotrial). As we have 90 total trials, we are only able to create four full chunks of 20 trials (90/(10 × 2) = 4.5, rounded down to 4 possible chunks). In this case, a random selection of trials would be left out on every iteration. We now create pseudotrials, using 10 of these 20 trials per average, creating two unique pseudotrials per chunk. See Figure 1C for another example of the leave‐n‐pseudotrials‐out procedure using both averaging and resampling.

In Experiment 1 (90 trials/condition), for both methods we resampled each trial between 1 and 15 times and used between 1 and 30 of the available trials per pseudotrial. In Experiment 2 (45 trials/condition), we resampled each trial between 1 and 10 times and used between 1 and 15 of the available trials per pseudotrial. Any trials that could not be used in a particular iteration of pseudotrial creation (this happened when the number of trials was not perfectly divisible by the number of trials per pseudotrial) were left out of that iteration of the analysis. To ensure that the results were not dependent on the specific division of trials into pseudotrials (Goddard et al. 2022), for each ‘subject’ and parameter set, we ran 100 iterations of the pseudotrial procedure and averaged the 100 resulting classification accuracies to give a single value for that subject and parameter set. This repetitive iteration of pseudotrial creation reduced the additional variance introduced by the averaging procedure and therefore appears to be essential to derive benefit from the pseudotrial scheme (see Figure S9 where we present results using different numbers of iterations). For all simulations, we report the average decoding accuracy across subjects, the standard deviation and the *t*‐statistic. This was calculated at the group‐level by subtracting 50 (theoretical chance level) and dividing by the standard error. In addition, we implemented a sign‐flip permutation test (cosmo montecarlo stat) for each parameter set. For each test we generated 10,000 permutations. Z‐score values greater than 1.96 indicate that the mean decoding was significantly different from the permuted null distribution at alpha = 0.05. We did not apply a multiple‐comparisons correction because each decoding result reflects an independent simulated experiment rather than repeated tests on the same data; however, this may still increase the risk of false‐positive findings. We did not implement a full permutation test for our large datasets given the extensive computation required to do so.

### 2.2 Simulation 2

Next, we ran a second set of simulations using the SEREEGA toolbox (Krol et al. 2018) to demonstrate the influence of averaging and resampling on more realistic simulated EEG data. We used the ICBM‐NY leadfield to simulate a 64‐channel montage in the 10–20 system. We generated a P300‐like central parietal ERP component with two conditions (although unlike a standard oddball paradigm with fewer targets than standards, here we simulated 90 trials for both conditions). Both had a peak latency of 350 ms, a wide peak width of 350 ms, with a different amplitude per condition. We chose a midbrain source that resulted in a classic central parietal topography when projected to the scalp (location = 0, −40, −25, orientation = 0.03, −0.16, 1). Epochs were generated at a sampling rate of 250 Hz and length of 600 ms. To simulate no difference in conditions, both peaks were given an amplitude of 11 mVs, defined at the source level. For a small effect, one amplitude was set to 10.5 mVs and the other amplitude was set to 11 mVs. For a large effect we used amplitudes of 10 mVs and 11 mVs. We also created a noise dataset that was generated using 25 random sources of white noise. These were mixed with the ERP data using a signal‐to‐noise ratio of 1/3. These amplitudes were chosen as the decoding accuracies from a standard analysis (with no averaging or resampling) were similar to the corresponding analysis performed on our first simulation. We acknowledge that there are a large number of parameters that could be varied in this simulation alone, but use this simple setup as an indicative example of an EEG dataset.

To emulate a more realistic EEG experiment, we simulated data for 30 participants (rather than 100 in the CoSMoMVPA simulations), the number of trials per condition was 90, the maximum number of trials per average was 15, the maximum resampling value was 10, and we used 50 iterations of random chunk allocation. Given the size of the computation required, we ran the decoding using SVM and Naïve Bayes classifiers only.

For simulation 2, we report the average decoding accuracy across subjects, the standard deviation and the *t*‐statistic. This was calculated at the group‐level by subtracting 50 (theoretical chance level) and dividing by the standard error. To mimic our other simulations, we report the average decoding across the epoch. Here, we implemented two versions of permutation testing, which we refer to as ‘full’ and ‘sign‐flip’ permutation. The sign‐flip permutation test was computed as before (using cosmo montecarlo stat). As the SEREEGA simulations were smaller than those run in CoSMoMVPA, we were also able to run full permutations. For the full permutation test, we created null distributions by randomly shuffling the condition labels in the data and recomputing the decoding accuracy 100 times (separately for each parameter set). For both versions of the permutation testing, Z‐score values greater than 1.96 indicate that the mean decoding was significantly different to the permuted null distribution at alpha = 0.05. While there was some variation in *Z* values for the data with no simulated difference, both versions correctly identified no significant effects across all parameters (i.e., there were no false positives with either method). For the other two effects (small and large), both permutation methods produced the same *Z* scores.

## 3 Results

### 3.1 Simulation 1: CoSMoMVPA

#### 3.1.1 Influence of Trial Averaging

**FIGURE 2:**
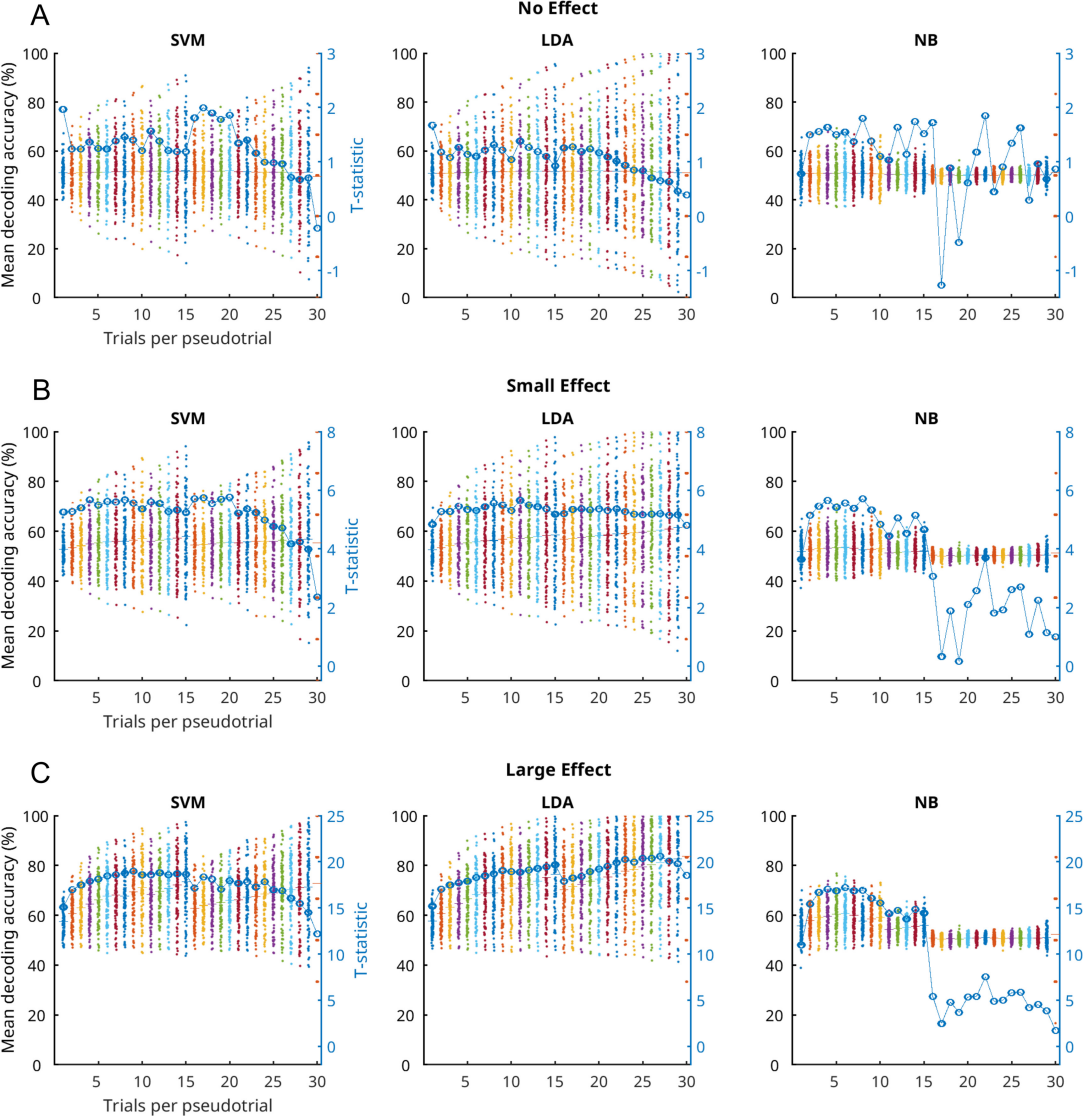
The influence of trial averaging, size of simulated effect and classifier type on decoding accuracy and *t*‐statistics. Rows correspond to the three simulated effects; (A) no effect, (B) small effect and (C) large effect. Columns correspond to results from the three classifiers tested (SVM = support vector machine, LDA = linear discriminant analysis, NB = Naïve Bayes). We simulated data from 100 subjects with 90 trials per condition. Pseudotrials were created separately within three ‘blocks’ of trials, facilitating a 3‐fold cross‐validation approach, with trials randomly allocated to pseudotrials across 100 iterations of pseudotrial creation for each subject. ‘Trials per pseudotrial’ indicates the number of trials used to create pseudotrials for each of the two simulated conditions, which was always matched. Using one trial per pseudotrial (the first value on the x‐axis) is equivalent to no trial averaging and using all 30 individual trials per condition per ‘block.’ Each datapoint represents the average cross‐validated decoding accuracy for one ‘subject.’ The *t*‐statistic (blue line and scale bar) is to quantify the trade‐off between modulating the mean decoding accuracy and variance over ‘subjects.’ It was calculated at the group‐level by subtracting 50 (theoretical chance level) and dividing by the standard error. Note that the *t*‐statistic range varies by effect size to improve visualisation.

First, we considered the influence of trial averaging for the data simulated in CoSMoMVPA. We used a 3‐fold cross‐validation procedure, in which trials were randomly allocated to one of three blocks before pseudotrials were created. Every analysis used the same 3‐fold cross validation procedure, and only the number of pseudotrials per block varied across averaging and resampling values. Importantly, for the dataset with no simulated difference between conditions (Figure 2A) average decoding accuracy remained around 50% with the creation of pseudotrials. This reassures us that averaging the data cannot create effects where they are not present in the underlying data.

For the data with small and large differences between conditions (Figure 2B,C), we found that averaging even a few trials together was beneficial for classification performance, resulting in increased decoding accuracy and higher *t*‐statistics, despite the concurrent increase in standard deviation. However, optimising this was nontrivial. Instead, there was a complex pattern of results that varied with the number of trials per pseudotrial, classifier type and size of effect. Including too many of the available trials per chunk in an average (reducing the number of datapoints available to the classifier) could be detrimental, as the increase in standard deviation outstripped the increase in average decoding, resulting in reduced *t* values. Once more than half of the available trials per chunk were used per average (more than 15 out of the 30 in each chunk), there was a decrease in the standard deviation across ‘subjects.’ This may be because the total number of pseudotrials than could be made was reduced to 1 (30/16 = 1.88, rounded down to 1). When there are fewer possible unique pseudotrials that can be created, the number of possible decoding outcome values across iterations will also be reduced. Using more than half of the available trials in a chunk per pseudotrial was particularly detrimental for the Naïve Bayes classifier, which benefited from less averaging and more samples for the classifier. This was also the case for the SVM classifier, but to a lesser extent, and most visible when the class distance was the greatest.

#### 3.1.2 Influence of Trial Resampling

Next, we examined the influence of trial resampling and its interaction with the number of trials per pseudotrial. Once again, with no simulated effect, decoding did not differ significantly from 50%, reassuring us that averaging and resampling the data cannot create effects where there are none (Figure S1). There were four false positives with the Naïve Bayes classifier (out of a total of 450 parameters), which arose when using a high number of trials per average (> 26). However, we generally recommend using a much smaller number of trials per average. Note that we did not apply a correction for multiple comparisons as each decoding result reflects an independent simulated experiment rather than repeated tests on the same data; however, this may still increase the risk of false‐positive findings. This very low number of false positives is within what we would expect to arise by chance when performing so many tests, rather than reflecting an effect of the pseudotrial method.

**FIGURE 3:**
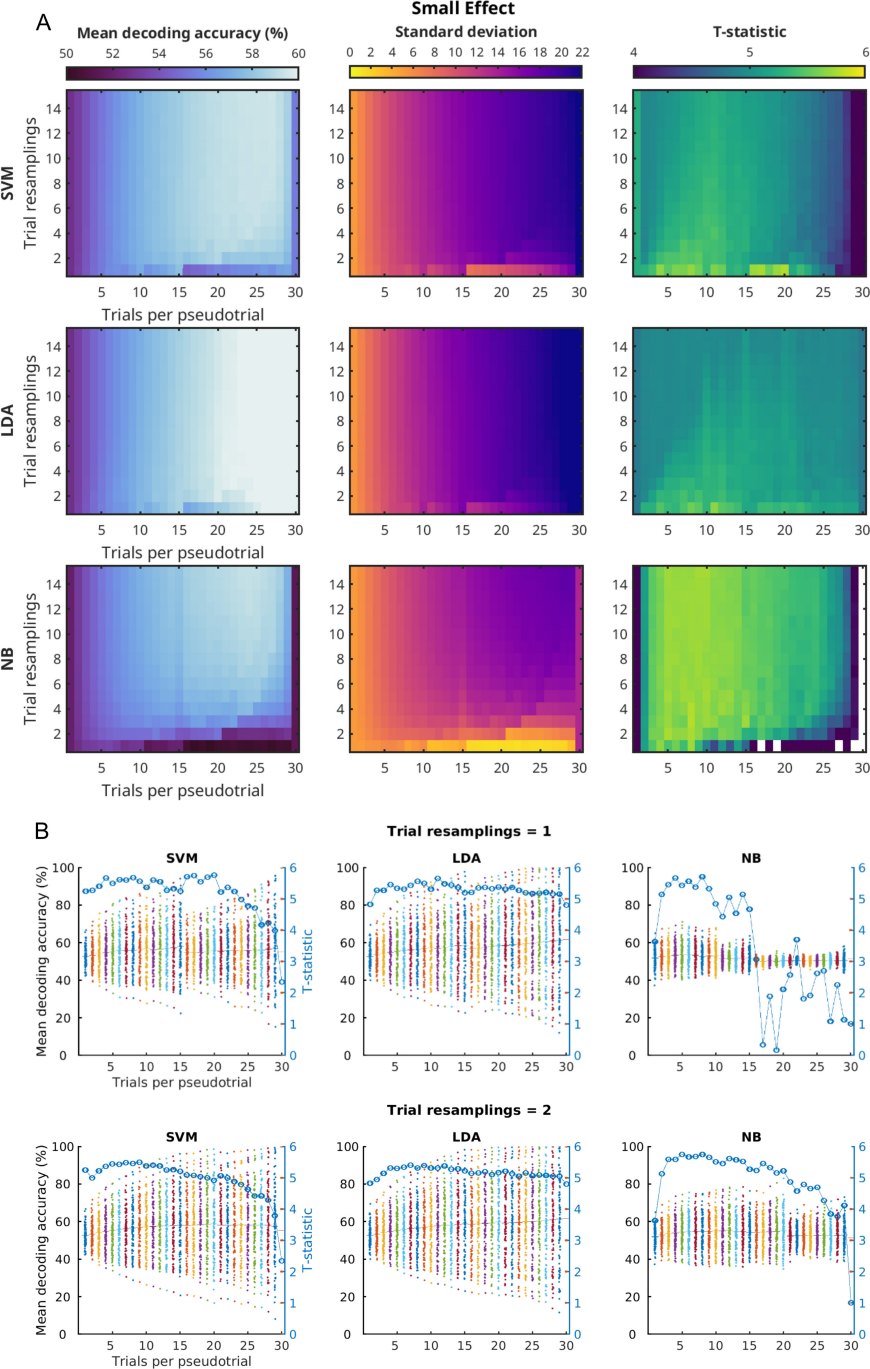
(A) The influence of averaging and resampling on decoding accuracy (left column), standard deviation across subjects (middle column) and group‐level *t*‐statistics (right column). Each cell represents the outcome across subjects for one combination of trial averaging and resampling values. Lighter colours on all three metrics correspond to a better decoding outcome (i.e., higher decoding values, lower standard deviation and higher *t*‐statistics). Results that were not significantly different from the permuted null using a sign‐flip permutation test are masked out from the *t*‐statistic plots (shown in white). Rows correspond to results from the three classifiers tested (SVM = support vector machine, LDA = linear discriminant analysis, NB = Naïve Bayes). Pseudotrials were created separately within three allocated ‘blocks’ of trials, facilitating a 3‐fold cross‐validation approach, with trials randomly allocated across 100 iterations of pseudotrial creation. For the results plotted here, we simulated data from 100 subjects with 90 trials per condition and a small effect between condition (see Figures S1 and S2 for the results with no effect and a large effect). (B) To further illustrate the influence of trial resampling value on decoding outcomes, we also show the scatter plots for resampling values of 1 (first row, reproduced from Figure 2B) and resampling of 2 (second row). These correspond to the data in the bottom two rows of the plots shown in 3A. Each datapoint represents the cross‐validated decoding accuracy for one subject. The *t*‐statistic was calculated at the group‐level to test the decoding accuracy against chance across all subjects (50%).

For the data with a small simulated difference between conditions (Figure 3), we found that using up to a third of the original trials per chunk to create each pseudotrial was sufficient to aid decoding (i.e., 10 of the 30 trials per chunk, or 11% of the total 90 trials). Combining this with a small amount of resampling further increased decoding accuracy, presumably because resampling allows more pseudotrials to be created. For example, with 30 trials per condition in each of the three chunks, averaging together 10 trials creates three pseudotrials per chunk if no resampling is used. With a resampling value of 2, each trial is included in two pseudotrials, meaning that six pseudotrials are created per chunk. While a resampling value of 2 increased *t* values for the Naïve Bayes classifier, this was not the case for SVM and LDA, where resampling mostly acted to stabilise the influence of the number of trials per pseudotrial. The results for a large effect were similar and can be found in Figure S2.

#### 3.1.3 Influence of Fewer Trials Per Condition

**FIGURE 4:**
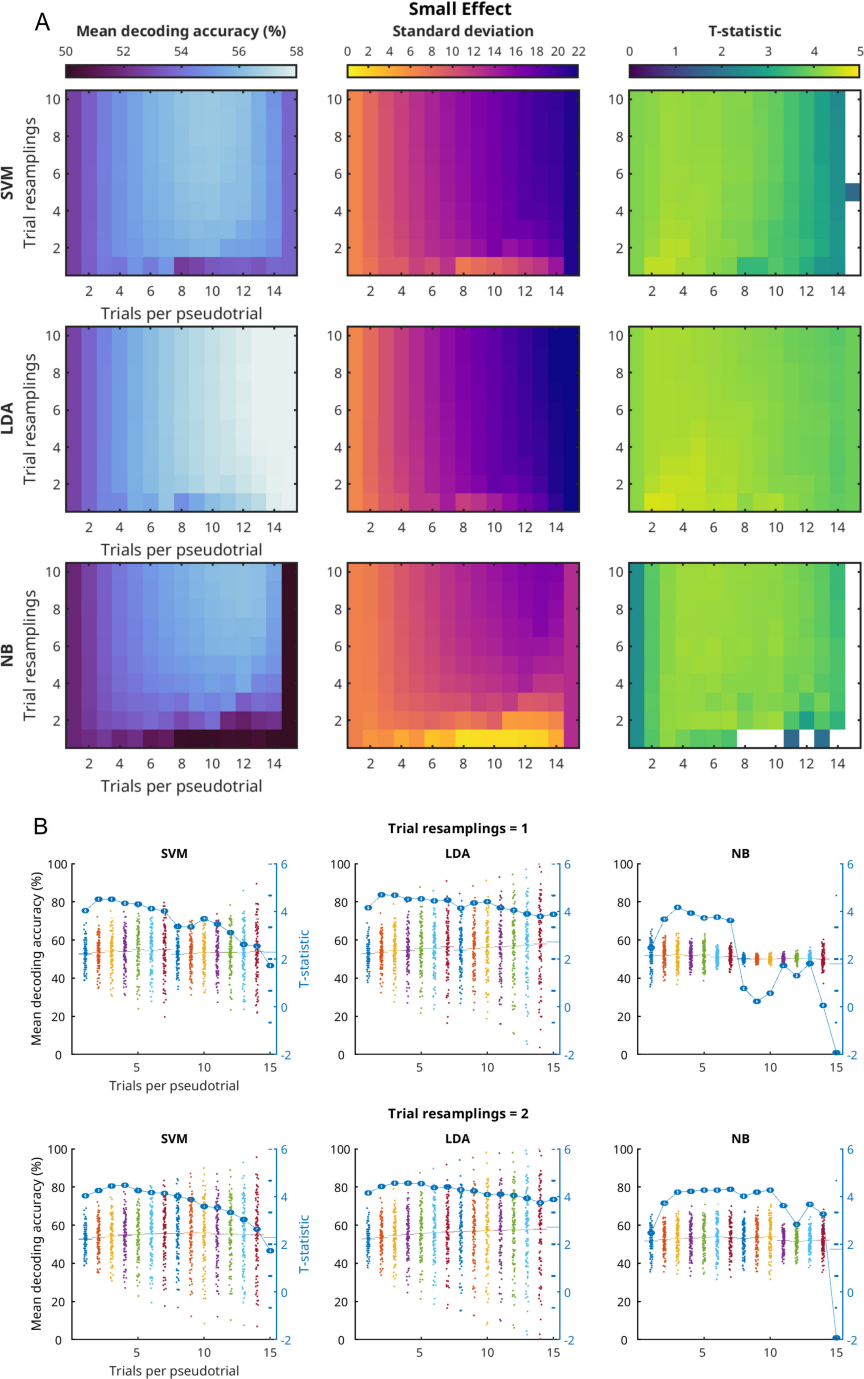
(A) The influence of averaging and resampling on decoding accuracy (left column), standard deviation across subjects (middle column) and group‐level *t*‐statistics (right column). Each cell represents the outcome across subjects for one combination of trial averaging and resampling values. Lighter colours on all three metrics correspond to a better decoding outcome (i.e., higher decoding values, lower standard deviation and higher *t*‐statistics). Results that were not significantly different from the permuted null using a sign‐flip permutation test are masked out from the *t*‐statistic plots (shown in white). Rows correspond to results from the three classifiers tested (SVM = support vector machine, LDA = linear discriminant analysis, NB = Naïve Bayes). Pseudotrials were created separately within three allocated ‘blocks’ of trials, facilitating a 3‐fold cross‐validation approach, with trials randomly allocated across 100 iterations of pseudotrial creation. For the results plotted here, we simulated data from 100 subjects with 45 trials per condition and a small effect (see Figure S3 for a large effect). (B) To further illustrate the influence of trial resampling value on decoding outcomes, we also show the scatter plots for resampling values of 1 and 2. These correspond to the data in rows 1 and 2 of the plots shown in A. Each datapoint represents the cross‐validated decoding accuracy for one subject. The *t*‐statistic was calculated at the group‐level to test the decoding accuracy against chance across all subjects (50%).

Next, we checked whether the same principles would apply to an experiment with fewer trials per condition. Figure 4 demonstrates the effect of trial resampling on a smaller dataset with only 45 trials per condition, meaning 15 trials in each of the three chunks. Once again, averaging up to a third of the original trials per chunk (i.e., 5 trials or less) to create each pseudotrial was sufficient to aid decoding performance. A small amount of resampling combined with trial averaging aided the classification particularly for the Naïve Bayes classifier (Figure 4B). However, high values on either parameter were generally detrimental for classifier performance; although the decoding accuracies were raised, an increase in standard deviation resulted in a decrease in *t* values (Figure 4A, column 3). The results for a large effect were similar and can be found in Figure S3.

#### 3.1.4 Influence of a ‘Leave‐n‐Pseudotrials‐Out’ Decoding Approach

Next, we examined the alternative ‘leave‐n‐pseudotrials‐out’ procedure, where the number of folds was determined by the number of pseudotrials created (number of folds = total trial number/(trials per average × trial resampling)). With no simulated effect, decoding remained mostly around 50%, reassuring us that averaging and resampling the data cannot create effects where there are none (Figure S4). However, there were two false positive findings for the SVM classifier when using eight or 17 trials per average with no resampling. In addition, there were five false positive findings for the Naïve Bayes classifier when using between four and eight trials per average. Note again that since we did not correct for multiple comparisons across tests, this level of false positives is within what we would expect to see as a result of running so many tests. See Figure S5 for results with a simulated effect size of 0.2.

**FIGURE 5:**
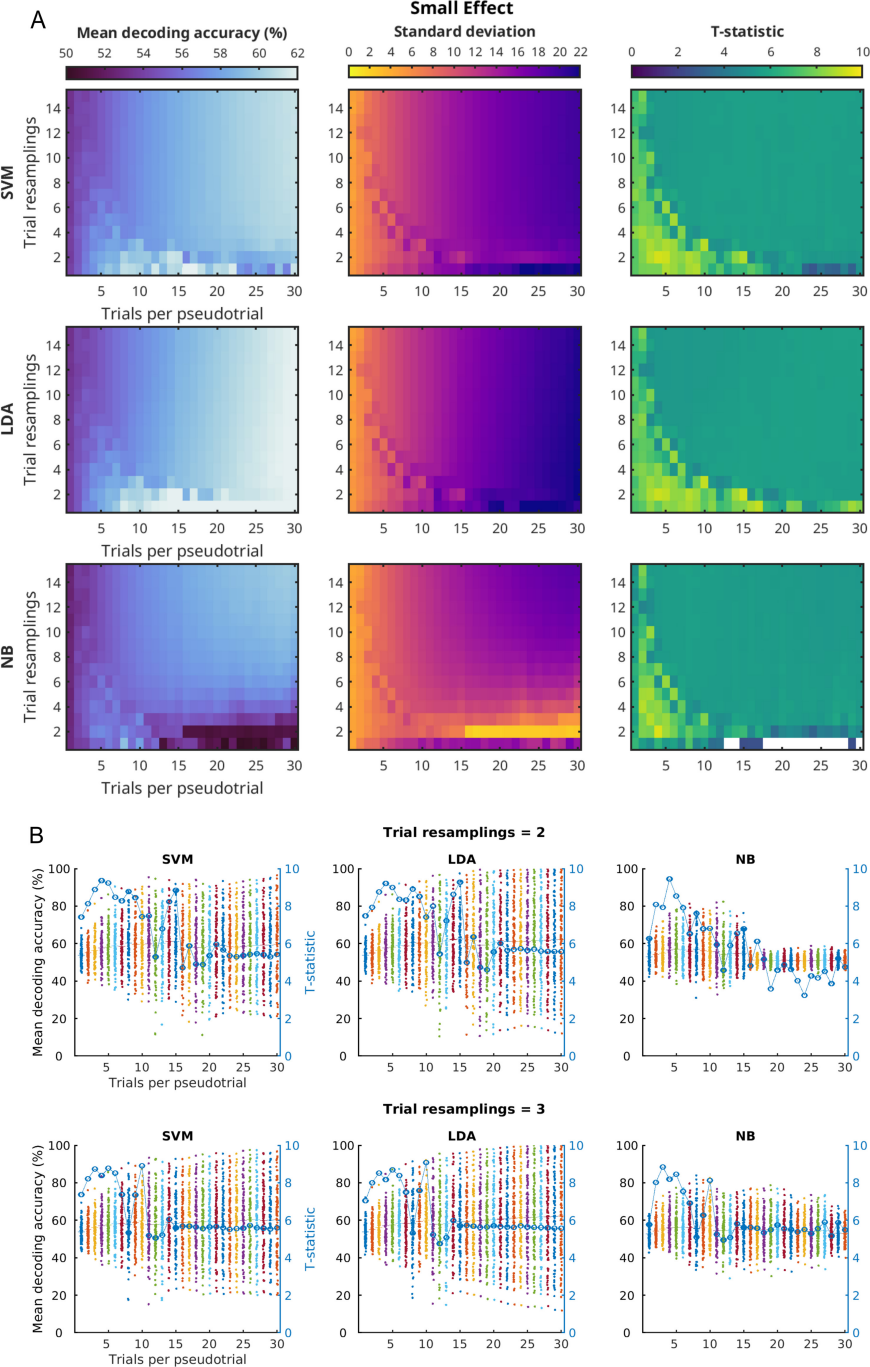
(A) The influence of averaging and resampling using a ‘leave‐n‐pseudotrials‐out’ approach on decoding accuracy (left column), standard deviation across subjects (middle column) and group‐level *t*‐statistics (right column). Each cell represents the outcome across subjects for one combination of trial averaging and resampling values. Lighter colours on all three metrics correspond to a better decoding outcome (i.e., higher decoding values, lower standard deviation and higher *t*‐statistics). Results that were not significantly different from the permuted null using a sign‐flip permutation test are masked out from the *t*‐statistic plots (shown in white). Rows correspond to results from the three classifiers tested (SVM = support vector machine, LDA = linear discriminant analysis, NB = Naïve Bayes). Here, the number of cross‐validation folds was determined by the number of trials per pseudotrial (the more trial averaging and resampling, the fewer possible folds), with trials randomly allocated across 100 iterations of pseudotrial creation. For the results plotted here, we simulated data from 100 subjects with 90 trials per condition and a small effect (see Figures S4 and S5 for the results with no effect and a large effect). (B) To further illustrate the influence of trial resampling value on decoding outcomes, we also show the scatter plots for resampling values of 2 and 3. These correspond to the data in rows 2 and 3 of the plots shown in A. Each datapoint represents the cross‐validated decoding accuracy for one subject. The *t*‐statistic was calculated at the group‐level to test the decoding accuracy against chance across all subjects (50%).

For the data with a small simulated difference between conditions, as shown in Figure 5, the largest *t*‐statistics were found across all classifiers when using roughly 5% of the total 90 trials per pseudotrial (i.e., 4 or 5 trials) with a resampling of 2. Therefore, fewer trials per average were necessary to aid classification in the ‘leave‐n‐pseudotrials‐out’ approach, compared to the optimal 11% for the three‐chunk version. The maximum decoding accuracies and *t* values achieved using the ‘n‐pseudotrials‐out’ method were also slightly higher overall than the ‘three‐chunk’ procedure, but had more variation across the parameter space. This is presumably due to the higher number of folds that could be used at low values of averaging and resampling. However, once the number of folds reached the minimum of 2, the influence of the parameters was reduced. This occurred when the combined averaging and resampling parameters exceed the number of trials that could be allocated to more than 2 folds.

### 3.2 Simulation 2: SEREEGA

#### 3.2.1 Influence of Simulation Type

**FIGURE 6:**
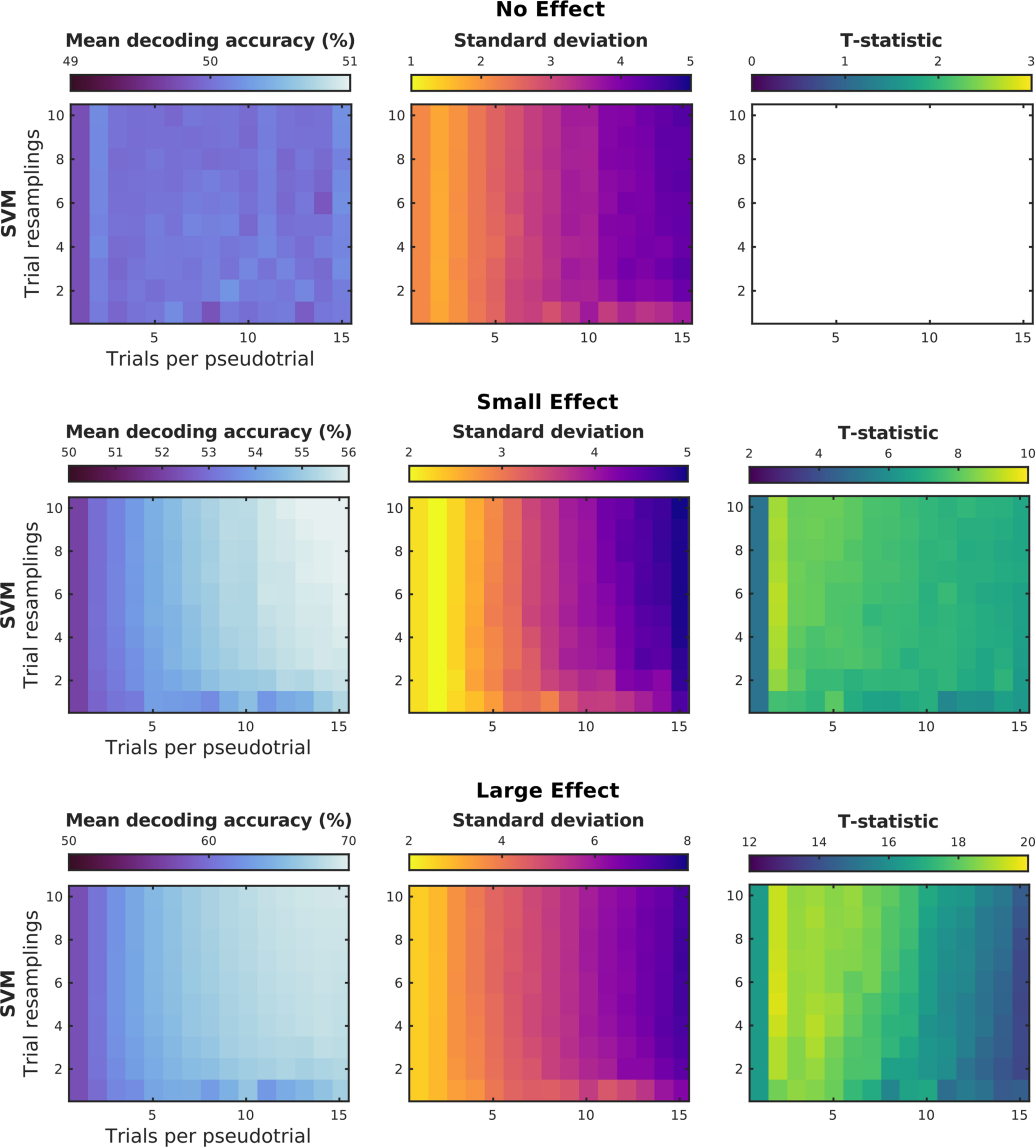
The influence of averaging and resampling using EEG data simulated using SEREEGA and an SVM classifier (support vector machine). We plot decoding accuracy (left column), standard deviation across subjects (middle column) and group‐level *t*‐statistics (right column). Each cell represents the outcome across subjects for one combination of trial averaging and resampling values. Lighter colours on all three metrics correspond to a better decoding outcome (i.e., higher decoding values, lower standard deviation and higher *t*‐statistics). Results that were not significantly different from the permuted null using a sign‐flip permutation test are masked out from the *t*‐statistic plots (shown in white). Rows correspond to results from the three effects tested (no effect, small effect and large effect). Note that the ranges of the plots vary by effect size to improve visualisation. Pseudotrials were created separately within three allocated ‘blocks’ of trials, facilitating a 3‐fold cross‐validation approach, with trials randomly allocated across 50 iterations of pseudotrial creation. For the results plotted here, we simulated data from 30 subjects with 90 trials per condition.

Next, we tested the influence of trial averaging and resampling on EEG data simulated in the SEREEGA toolbox. These simulations should more closely resemble real EEG data and provide another test for these parameter choices. We chose to simulate a central parietal P300‐like ERP component with two conditions. For comparison with our other results, we averaged the decoding outcome across time. Figure 6 shows the decoding results for an SVM classifier with three different effect sizes. The results were similar to the previous simulations. When we simulated an ERP with the same amplitude in each condition (no effect), decoding remained close to 50% across all parameters, and none of the permutation tests indicated a significant effect (i.e., there were no false positives). For both a small and large simulated difference between conditions, a small amount of averaging was sufficient to boost decoding accuracies and their associated *t* values (approximately 2–4 trials per average, corresponding to 7%–13% of the 30 trials per chunk, or 2%–4% of the total 90 trials per condition). Using too many trials per average increased the standard deviation across ‘subjects’ and subsequently reduced the *t*‐statistics.

**FIGURE 7:**
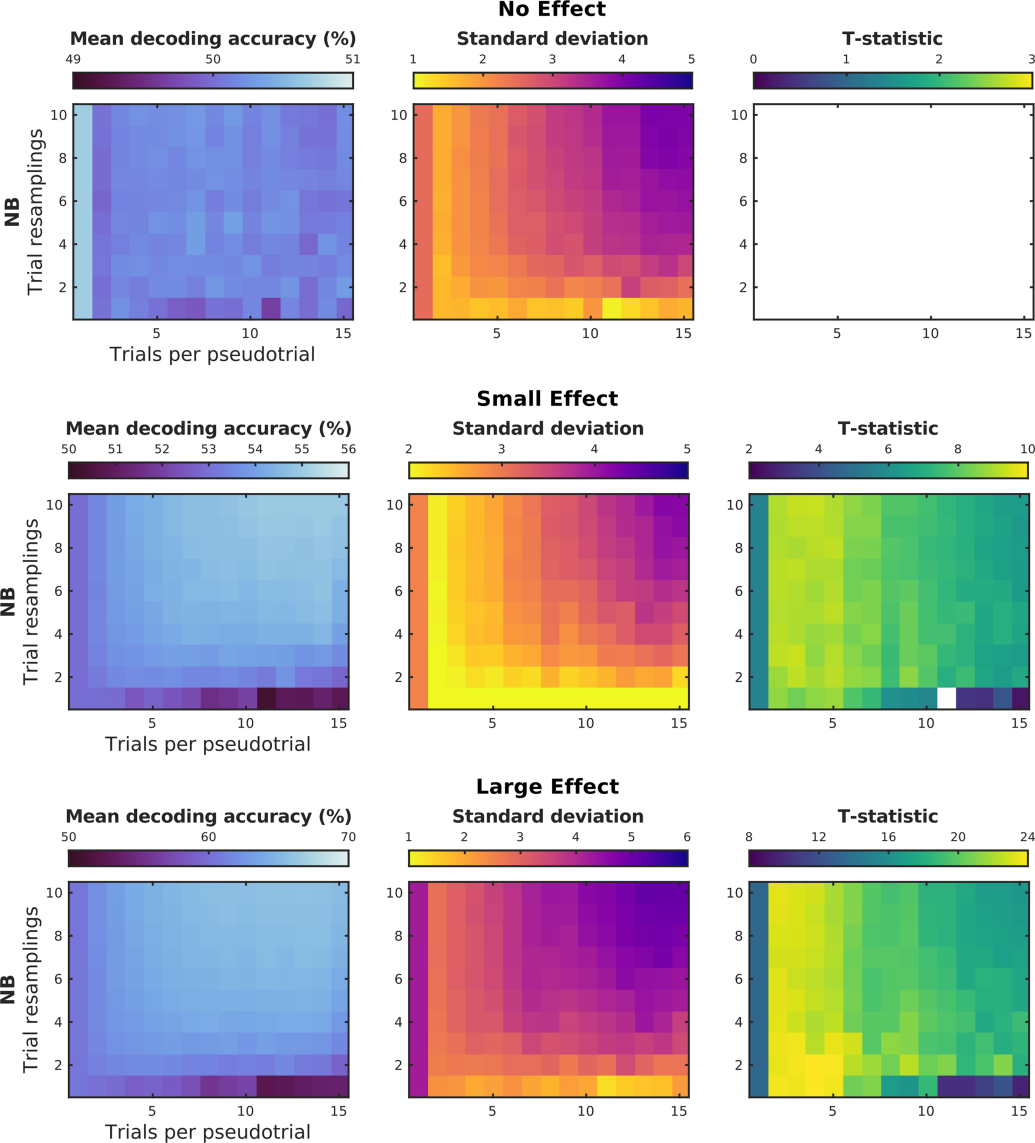
The influence of averaging and resampling using EEG data simulated using SEREEGA and a Naïve Bayes classifier (NB). We plot decoding accuracy (left column), standard deviation across subjects (middle column) and group‐level *t*‐statistics (right column). Each cell represents the outcome across subjects for one combination of trial averaging and resampling values. Lighter colours on all three metrics correspond to a better decoding outcome (i.e., higher decoding values, lower standard deviation and higher *t*‐statistics). Results that were not significantly different from the permuted null using a sign‐flip permutation test are masked out from the *t*‐statistic plots (shown in white). Rows correspond to results from the three effects tested (no effect, small effect and large effect). Note that the ranges of the plots vary by effect size to improve visualisation. Pseudotrials were created separately within three allocated ‘blocks’ of trials, facilitating a 3‐fold cross‐validation approach, with trials randomly allocated across 50 iterations of pseudotrial creation. For the results plotted here, we simulated data from 30 subjects with 90 trials per condition.

Figure 7 shows the equivalent decoding results instead using a Naïve Bayes classifier. When we simulated an ERP with the same amplitude in each condition (no effect), decoding remained close to 50% across all parameters, and none of the permutation tests indicated a significant effect (i.e., there were no false positives). For both a small and large simulated difference between conditions, a small amount of averaging was sufficient to boost decoding accuracies and their associated *t* values (approximately 2–5 trials per average, corresponding to 7%–16% of the 30 trials per chunk, or 2%–6% of the total 90 trials per condition). Again, using too many trials per average increased the standard deviation across ‘subjects’ and subsequently reduced the *t*‐statistics. As we saw in our other simulations, Naïve Bayes performed poorly once more than half of the available trials per chunk were used to create pseudotrials (if no resampling was used). Overall, the performance of both classifiers was similar across our two simulations, despite the differences between the simulated signals.

## 4 Discussion

We examined the influence of averaging and resampling on decoding outcomes across several parameters, including classifier type, size of simulated effect, number of trials available per condition and the cross‐validation procedure. We simulated data in two different toolboxes (CoSMoMVPA and SEREEGA), which vary in their complexity and similarity to real EEG data. Across both simulations, increasing the number of trials used per pseudotrial generally increased decoding accuracy, but also increased the between‐subject variance. We used a *t*‐test to quantify the trade‐off between these factors and found that, with a ‘three‐chunk’ or ‘10‐chunk’ approach, using up to a third of the original number of trials per chunk (or ~10% of all trials for 3‐chunk, ~3% of all trials for 10‐chunk) aided decoding. This was consistent for both experiment sizes. For the three‐chunk procedure, there was an additional stabilising effect provided by a low resampling value of 2. This was equivalent to creating six pseudotrials within each of the three chunks (18 pseudotrials in total). However, too much averaging could be detrimental, particularly for the Naïve Bayes classifier, and little was gained by using high resampling values except to compensate for too much averaging using Naïve Bayes.

For the ‘leave‐n‐pseudotrials‐out’ cross‐validation approach, fewer trials were needed per pseudotrial to optimise the decoding outcome. Using around a sixth of the original number of trials per chunk (or 5% of all trials) produced the largest *t*‐statistics across all classifiers when combined with a low resampling value of 2. Higher decoding accuracies and *t*‐statistics were achieved for some parameters using the ‘leave‐n‐pseudotrials‐out’ approach compared to the ‘three‐chunk’ version, but the different number of decoding folds resulted in more variation across the parameter space.

Although there were similarities across the three classifiers used, they responded differently across the parameter space, presumably due to the differences in their functions. Linear SVM separates classes by positioning a decision hyperplane in pattern space (Misaki et al. 2010). This hyperplane is chosen by maximising the distance to the patterns on either side, using the most informative data points that lie closest to the decision boundary (support vectors). Because of this, the SVM is not as influenced by changes in data points sitting away from the decision boundary. Therefore, SVM can perform well with limited data and will benefit most from having a few stable estimates near the decision boundary (Mur et al. 2009). Averaging may therefore aid classification using SVM by stabilising the data used to position the hyperplane.

In LDA, the pattern space is constructed by maximising the between‐class variance while minimising the within‐class variance. The hyperplane is positioned in the middle of the class means, assuming that the two classes have Gaussian distributions and equal covariance. Therefore, a change in any data point will shift the decision boundary and potentially influence the classification result. However, when the distributions are relatively normal and there are no extreme outliers (as is the case here), SVM and LDA are likely to perform similarly. Gaussian Naïve Bayes is similar to LDA, but also assumes that there are no correlations between pairs of data points within the same class (zero off‐diagonal covariance). Having a low number of data points in each class distribution may more negatively impact the performance of Gaussian Naïve Bayes (Misaki et al. 2010), as demonstrated here.

Here, we focused on *t*‐scores derived from decoding accuracy as a quantification of the trade‐off between increasing mean decoding and variance. Other metrics of class separation have been proposed, such as cross‐validated Mahalanobis distance (Walther et al. 2016). We believe that our results would generalise to such other measures, as our results show that averaging increases separation between datapoints, evidenced by increased accuracy, which would similarly increase Mahalanobis distance. However, a full exploration of the effect of averaging on these other measures is outside the scope of the current paper. We also acknowledge that real neuroimaging data is more complex than the data simulated here, but aim to provide a starting point for researchers to examine the effects of decoding parameters in the simplest case.

While we demonstrate averaging and resampling on simulated EEG data, our results can also inform fMRI analysis. When analysing fMRI data, the most common strategy is to estimate the activity for a given condition using all of the available trials in a run. This is akin to averaging trials within scanning runs, as the condition beta will reflect an approximate average of the activity across trials. This is a common approach, as estimating single‐trial activity in fMRI can be difficult given the noise in the signal. However, there is a trade‐off between including more trials per estimate of a regressor (modelling all trials of one condition per run together) and having more regressors per condition (at the extreme: modelling each individual trial as one regressor). The optimal balance between these choices for fMRI is likely to depend on the balance between condition variance and scan variance, which can be experiment specific. At the level of MVPA, however, the trade‐off is conceptually similar to that faced in EEG and explored here: the more trials that you allocate to an average, the fewer possible pseudotrials you can create per condition.

In summary, we found that modest trial averaging can improve decoding accuracy and associated *t*‐statistics and that a small amount of resampling helps to stabilise the benefit of doing so. However, only a low resampling value is helpful and is not always necessary. In addition, the use of pseudotrials did not increase decoding accuracies when no effect was present. Although we provide general guidelines, the optimal parameter choice (particularly, the number of trials per pseudotrial) will be data and design specific, so we provide analysis code for others to run simulations based on their own design and hypothesised effects. These analyses show that the details of trial averaging and decoding scheme will affect the results a researcher is likely to find. Our hope is that these guidelines, together with code for targeted simulations, can aid researchers in deciding their decoding analysis scheme and parameters up front, so that they are not tempted to run multiple versions of analyses on real data. In this way our guidelines and code can be used to inform future multivariate brain decoding studies.

## Author Contributions

**Catriona L. Scrivener:** formal analysis, methodology, software, visualization, writing – original draft. **Tijl Grootswagers:** conceptualization, methodology, software, visualization, writing – review and editing. **Alexandra Woolgar:** conceptualization, funding acquisition, methodology, software, supervision, visualization, writing – review and editing.

## Funding

This work was supported by the Australian Research Council (DE230100380 and DP170101840), the Biotechnology and Biological Sciences Research Council (BB/V003887/1) and the Medical Research Council (MC_UU_00030/15).

## Ethics Statement

The author have nothing to report.

## Conflicts of Interest

The authors declare no conflicts of interest.

## Supporting information


**Figure S1:** The influence of both averaging and resampling, on decoding accuracy, standard deviation and *t*‐statistics when the simulated data has no underlying effect. Results that were not significantly different from the permuted null using a sign‐flip permutation test are masked out from the *t*‐statistic plots (shown in white). Note that in the case of no underlying effect, we expect to find a nonsignificant decoding result for all tests, and coloured points indicate false positives. Rows correspond to results from the three classifiers tested (SVM = support vector machine, LDA = linear discriminant analysis, NB = Naïve Bayes). Pseudotrials were created separately within three allocated ‘blocks’ of trials, facilitating a 3‐fold cross‐validation approach, with trials randomly allocated across 100 iterations of pseudotrial creation. For the results plotted here, we simulated data from 100 subjects with 90 trials per condition with no effects, or a class distance of 0 (see main Figure 3 for a small effect, or class distance of 0.1).
**Figure S2:** The influence of both averaging and resampling, on decoding accuracy, standard deviation and *t*‐statistics for simulated data with a large underlying effect. Results that were not significantly different from the permuted null using a sign‐flip permutation test are masked out from the *t*‐statistic plots (shown in white). Rows correspond to results from the three classifiers tested (SVM = support vector machine, LDA = linear discriminant analysis). Pseudotrials were created separately within three allocated ‘blocks’ of trials, facilitating a 3‐fold cross‐validation approach, with trials randomly allocated across 100 iterations of pseudotrial creation. For the results plotted here, we simulated data from 100 subjects with 90 trials per condition and a large effect, or a class distance of 0.2 (see main Figure 3 for a small effect, or a class distance of 0.1).
**Figure S3:** The influence of both averaging and resampling for a dataset with only 45 trials per condition for simulated data with a large underlying effect. Results that were not significantly different from the permuted null using a sign‐flip permutation test are masked out from the *t*‐statistic plots (shown in white). Rows correspond to results from the three classifiers tested (SVM = support vector machine, LDA = linear discriminant analysis, NB = Naïve Bayes). Pseudotrials were created separately within three allocated ‘blocks’ of trials, facilitating a 3‐fold cross‐validation approach, with trials randomly allocated across 100 iterations of pseudotrial creation. For the results plotted here, we simulated data from 100 subjects with 45 trials per condition and large effect, or a class distance of 0.2 (see main Figure 4 for a small effect, or a class distance of 0.1).
**Figure S4:** The influence of averaging and resampling using a ‘leave‐n‐pseudotrials‐out’ decoding approach for simulated data with no underlying effect. Results that were not significantly different from the permuted null using a sign‐flip permutation test are masked out from the *t*‐statistic plots (shown in white). Note that in the case of no underlying effect, we expect to find a nonsignificant decoding result for all tests, and coloured points indicate false positives. Rows correspond to results from the three classifiers tested (SVM = support vector machine, LDA = linear discriminant analysis, NB = Naïve Bayes). Here, the number of cross‐validation folds was determined by the number of trials per pseudotrial (the more trial averaging and resampling, the fewer possible folds), with trials randomly allocated across 100 iterations of pseudotrial creation. For the results plotted here, we simulated data from 100 subjects with 90 trials per condition and a large effect, or class distance 0 (see main Figure 5 for a small effect, or class distance of 0.1, and Figure S5 for a large effect, or class distance of 0.2).
**Figure S5:** The influence of averaging and resampling using a ‘leave‐n‐pseudotrials‐out’ decoding approach for simulated data with a large underlying effect. Results that were not significantly different from the permuted null using a sign‐flip permutation test are masked out from the *t*‐statistic plots (shown in white). Rows correspond to results from the three classifiers tested (SVM = support vector machine, LDA = linear discriminant analysis, NB = Naïve Bayes). Here, the number of cross‐validation folds was determined by the number of trials per pseudotrial (the more trial averaging and resampling, the fewer possible folds), with trials randomly allocated across 100 iterations of pseudotrial creation. For the results plotted here, we simulated data from 100 subjects with 90 trials per condition and a large effect, or class distance of 0.2 (see main Figure 5 for a small effect, or class distance of 0.1, and supplementary Figure 4 for no effect, or class distance of 0).
**Figure S6:** The influence of both averaging and resampling with fewer subjects (*n* = 50) for simulated data with a large underlying effect. Results that were not significantly different from the permuted null using a sign‐flip permutation test are masked out from the *t*‐statistic plots (shown in white). Rows correspond to results from the three classifiers tested (SVM = support vector machine, LDA = linear discriminant analysis, NB = Naïve Bayes). Pseudotrials were created separately within three allocated ‘blocks’ of trials, facilitating a 3‐fold cross‐validation approach, with trials randomly allocated across 100 iterations of pseudotrial creation. For the results plotted here, we simulated data from 50 subjects with 90 trials per condition and a large effect (class distance of 0.2).
**Figure S7:** The influence of both averaging and resampling with a smaller number of features (data size ‘small’ = 6 features) for simulated data with a large underlying effect. Results that were not significantly different from the permuted null using a sign‐flip permutation test are masked out from the *t*‐statistic plots (shown in white). Rows correspond to results from the three classifiers tested (SVM = support vector machine, LDA = linear discriminant analysis, NB = Naïve Bayes). Pseudotrials were created separately within three allocated ‘blocks’ of trials, facilitating a 3‐fold cross‐validation approach, with trials randomly allocated across 100 iterations of pseudotrial creation. For the results plotted here, we simulated data from 100 subjects with 90 trials per condition and a large effect (class distance of 0.2).
**Figure S8:** The influence of averaging and resampling using a higher number of cross‐validation folds for simulated data with a small underlying effect. Rows correspond to results from the three classifiers tested (SVM = support vector machine, LDA = linear discriminant analysis, NB = Naïve Bayes). Pseudotrials were created separately within 10 allocated ‘blocks’ of nine trials each, facilitating a 10‐fold cross‐validation approach, with trials randomly allocated across 100 iterations of pseudotrial creation. For the results plotted here, we simulated data from 100 subjects with 90 trials per condition and a small effect (class distance of 0.1).
**Figure S9:** The influence of fewer iterations of random trial allocation for simulated data with a large underlying effect. For the results plotted here, we simulated data from 100 subjects with 90 trials per condition and a large effect (class distance of 0.2). Rows correspond to the number of iterations of random trial allocation that was used to create pseudotrials. Columns correspond to results from the three classifiers tested (SVM = support vector machine, LDA = linear discriminant analysis, NB = Naïve Bayes). Pseudotrials were created separately within three allocated ‘blocks’ of trials, facilitating a 3‐fold cross‐validation approach, with trials randomly allocated.

## Data Availability

Analysis scripts can be found at https://osf.io/hjf75/.
