# Supplementary material for: Optimising Analysis Choices for Multivariate Decoding: Creating Pseudotrials Using Trial Averaging and Resampling
Source: Eur J Neurosci. 2026 Jul 19;64(2):e70601. doi: 10.1111/ejn.70601 (PMC13382189; doi:10.1111/ejn.70601)
Supplement: Supplementary file 1 — Figure S1: The influence of both averaging and resampling, on decoding accuracy, standard deviation and t‐statistics when the simulated data has no underlying effect. Results that were not significantly different from the permuted null using a sign‐flip permutation test are masked out from the t‐statistic plots (shown in white). Note that in the case of no underlying effect, we expect to find a nonsignificant decoding result for all tests, and coloured points indicate false positives. Rows correspond to results from the three classifiers tested (SVM = support vector machine, LDA = linear discriminant analysis, NB = Naïve Bayes). Pseudotrials were created separately within three allocated ‘blocks’ of trials, facilitating a 3‐fold cross‐validation approach, with trials randomly allocated across 100 iterations of pseudotrial creation. For the results plotted here, we simulated data from 100 subjects with 90 trials per condition with no effects, or a class distance of 0 (see main Figure 3 for a small effect, or class distance of 0.1). Figure S2: The influence of both averaging and resampling, on decoding accuracy, standard deviation and t‐statistics for simulated data with a large underlying effect. Results that were not significantly different from the permuted null using a sign‐flip permutation test are masked out from the t‐statistic plots (shown in white). Rows correspond to results from the three classifiers tested (SVM = support vector machine, LDA = linear discriminant analysis). Pseudotrials were created separately within three allocated ‘blocks’ of trials, facilitating a 3‐fold cross‐validation approach, with trials randomly allocated across 100 iterations of pseudotrial creation. For the results plotted here, we simulated data from 100 subjects with 90 trials per condition and a large effect, or a class distance of 0.2 (see main Figure 3 for a small effect, or a class distance of 0.1). Figure S3: The influence of both averaging and resampling for a dataset wit [file EJN-64-0-s001.docx]

**Optimising analysis choices for multivariate decoding: creating pseudotrials using trial averaging and resampling**

# Supplementary Material

Here we present additional results for effect sizes that were not included in the main text. This includes the influence of trial resampling*,* fewer trials per condition, and a leave-n-pseudotrials-out approach. We also present additional analyses that were not addressed in the main text. This includes the influence of a smaller number of subjects, a smaller number of features, increasing the number of cross-validation folds to 10, and using different iterations of random trial allocation to pseudotrials.

# S1. Influence of trial resampling (additional no effect and large effect)

Here we examined the influence of trial resampling on decoding accuracy when there was no simulated class difference. This differs from **main Figure 3** which displayed the results for a small effect (class distance of 0.1). Decoding performed on data with no simulated effect mostly remained around 50%, reassuring us that averaging and resampling the data cannot create effects where there is none (**supplementary Figure 1**). There were four false positives with the Naïve Bayes classifier (out of a total of 450 parameters), specifically when using a high number of trials per average (>26). However, we generally recommend using a much smaller number of trials per average. Note that we did not apply a correction for multiple comparisons as each decoding result reflects an independent simulated experiment rather than repeated tests on the same data; however, this may still increase the risk of false-positive findings. This very low number of false positives is within what we would expect to arise by chance when performing so many tests, rather than reflecting an effect of the pseudotrial method.


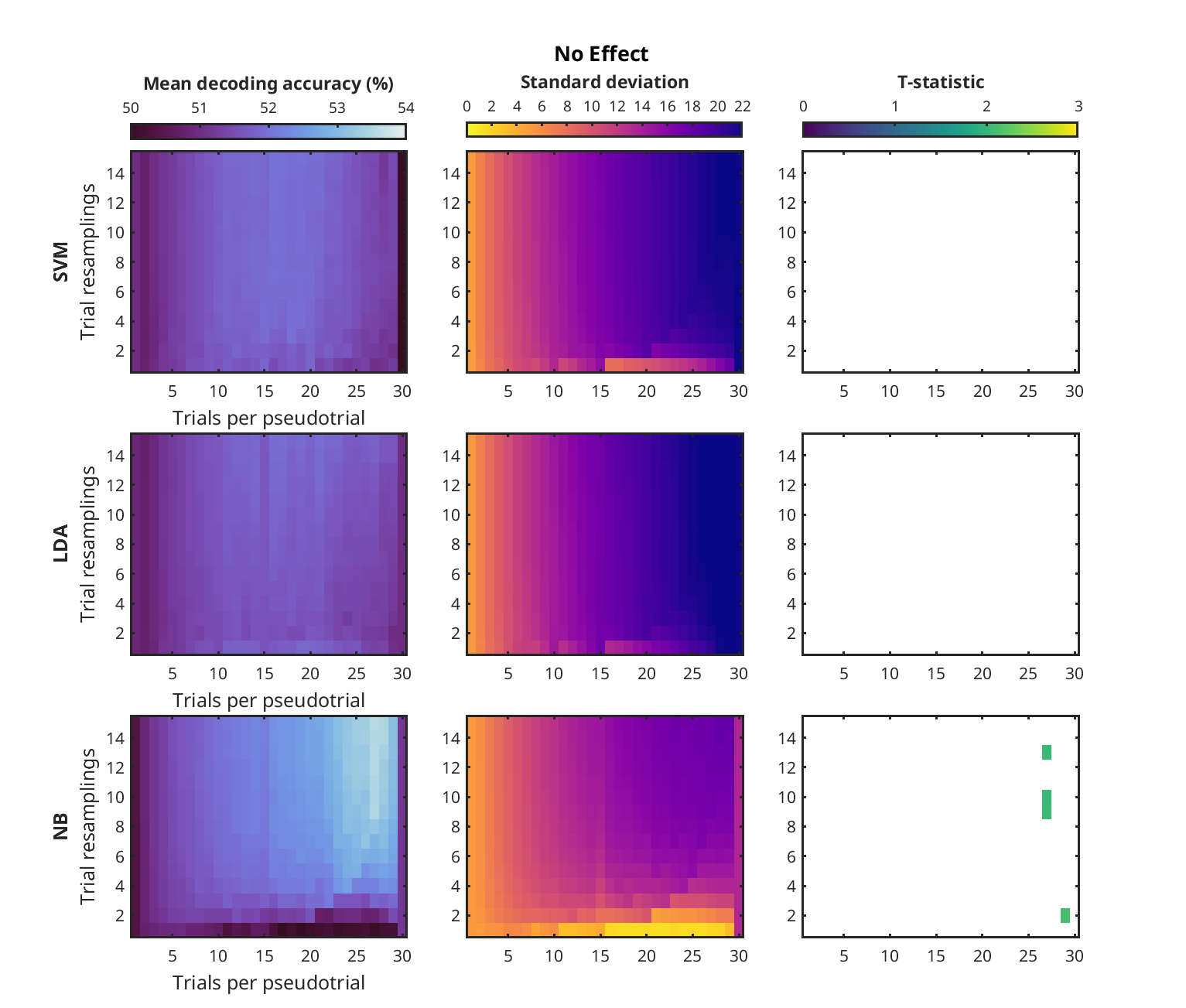


**Supplementary Figure 1.** The influence of both averaging and resampling, on decoding accuracy, standard deviation, and t-statistics when the simulated data has no underlying effect. Results that were not significantly different from the permuted null using a sign-flip permutation test are masked out from the t-statistic plots (shown in white). Note that in the case of no underlying effect, we expect to find a non-significant decoding result for all tests, and coloured points indicate false positives. Rows correspond to results from the three classifiers tested (SVM = support vector machine, LDA = linear discriminant analysis, NB = Naïve Bayes). Pseudotrials were created separately within 3 allocated ‘blocks’ of trials, facilitating a 3-fold cross-validation approach, with trials randomly allocated across 100 iterations of pseudotrial creation. For the results plotted here, we simulated data from 100 subjects with 90 trials per condition with no effects, or a class distance of 0 (see main Figure 3 for a small effect, or class distance of 0.1).


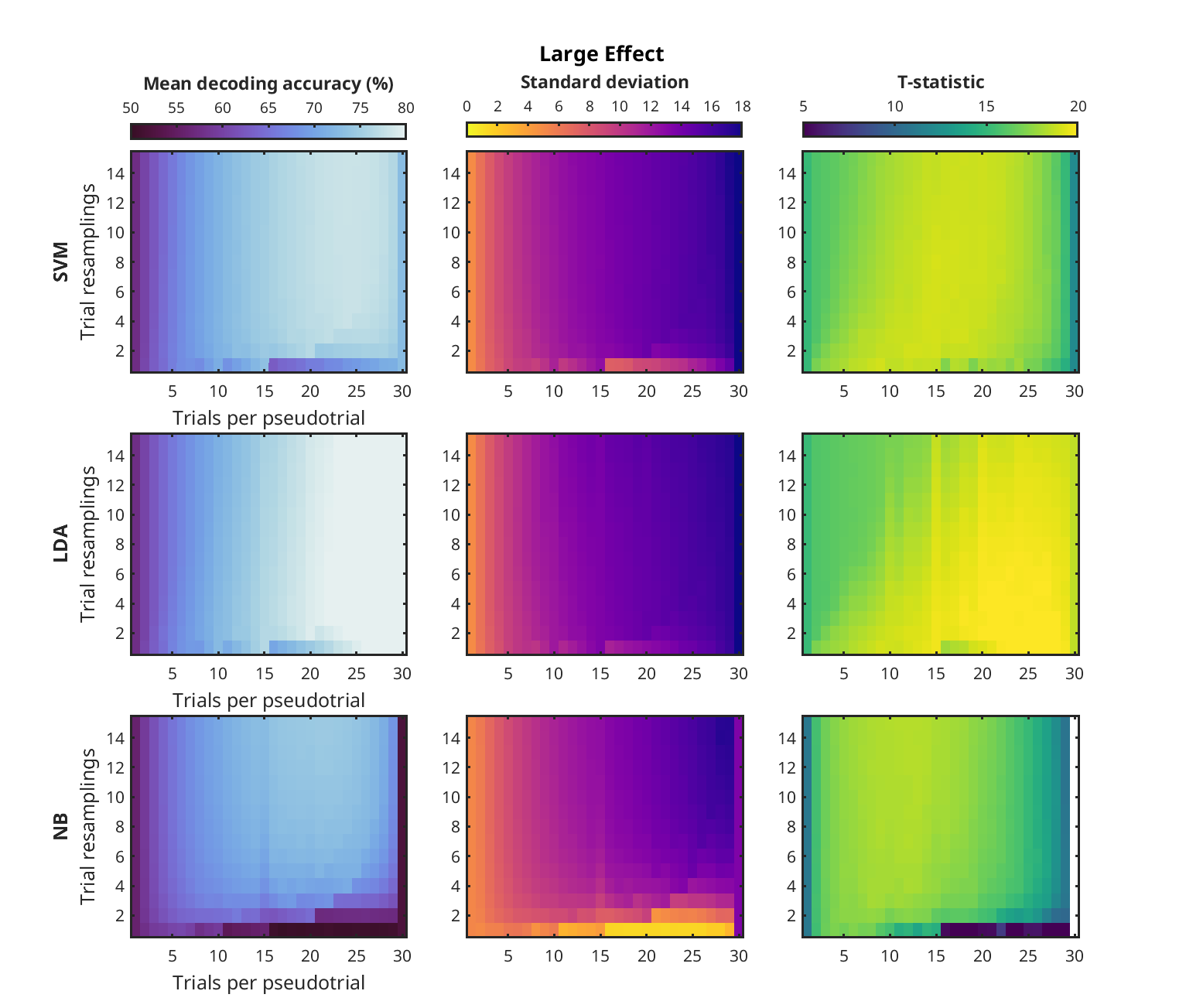
For the data with a small simulated difference between conditions of 0.1 (**main Figure 3**) we found that using around a third of the original trials per chunk to create each pseudotrial (i.e., 10 trials) was sufficient to aid decoding. Combining this with a small amount of resampling further increased decoding accuracy. As shown in **Supplementary Figure 2**, this was also true for the data with a large effect (a class distance of 0.2), when using the Naïve Bayes classifier. These parameters also performed well for the SVM and LDA classifiers, but SVM performed equally with up to 50% of the original trials per pseudotrial (i.e., 15 trials) and a resampling value of 2. The LDA classifier performed well across a large range of parameters, providing that the resampling value was not higher than the number of trials per pseudotrial. With the larger simulated effect, the classifiers appeared to be more robust to the choice of averaging and resampling parameters.

**Supplementary Figure 2.** The influence of both averaging and resampling, on decoding accuracy, standard deviation, and t-statistics for simulated data with a large underlying effect. Results that were not significantly different from the permuted null using a sign-flip permutation test are masked out from the t-statistic plots (shown in white). Rows correspond to results from the three classifiers tested (SVM = support vector machine, LDA = linear discriminant analysis). Pseudotrials were created separately within 3 allocated ‘blocks’ of trials, facilitating a 3-fold cross-validation approach, with trials randomly allocated across 100 iterations of pseudotrial creation. For the results plotted here, we simulated data from 100 subjects with 90 trials per condition and a large effect, or a class distance of 0.2 (see main Figure 3 for a small effect, or a class distance of 0.1).

# S2. Influence of fewer trials per condition (additional large effect)

Here we examined the effect of trial resampling on a smaller dataset with only 45 trials per condition, meaning 15 trials in each of the 3 chunks. This differs from **main Figure 4** which displayed the results for small effect (a class distance of 0.1). Once again, a small amount of resampling combined with trial averaging aided the classification. High values on either parameter was detrimental for classifier performance, and using up to a third of the original trials (i.e., 5 trials or less) per pseudotrial with a resampling of 2 was optimal for both a small effect (**main Figure 4**), and a large effect (**supplementary Figure 3**).

**
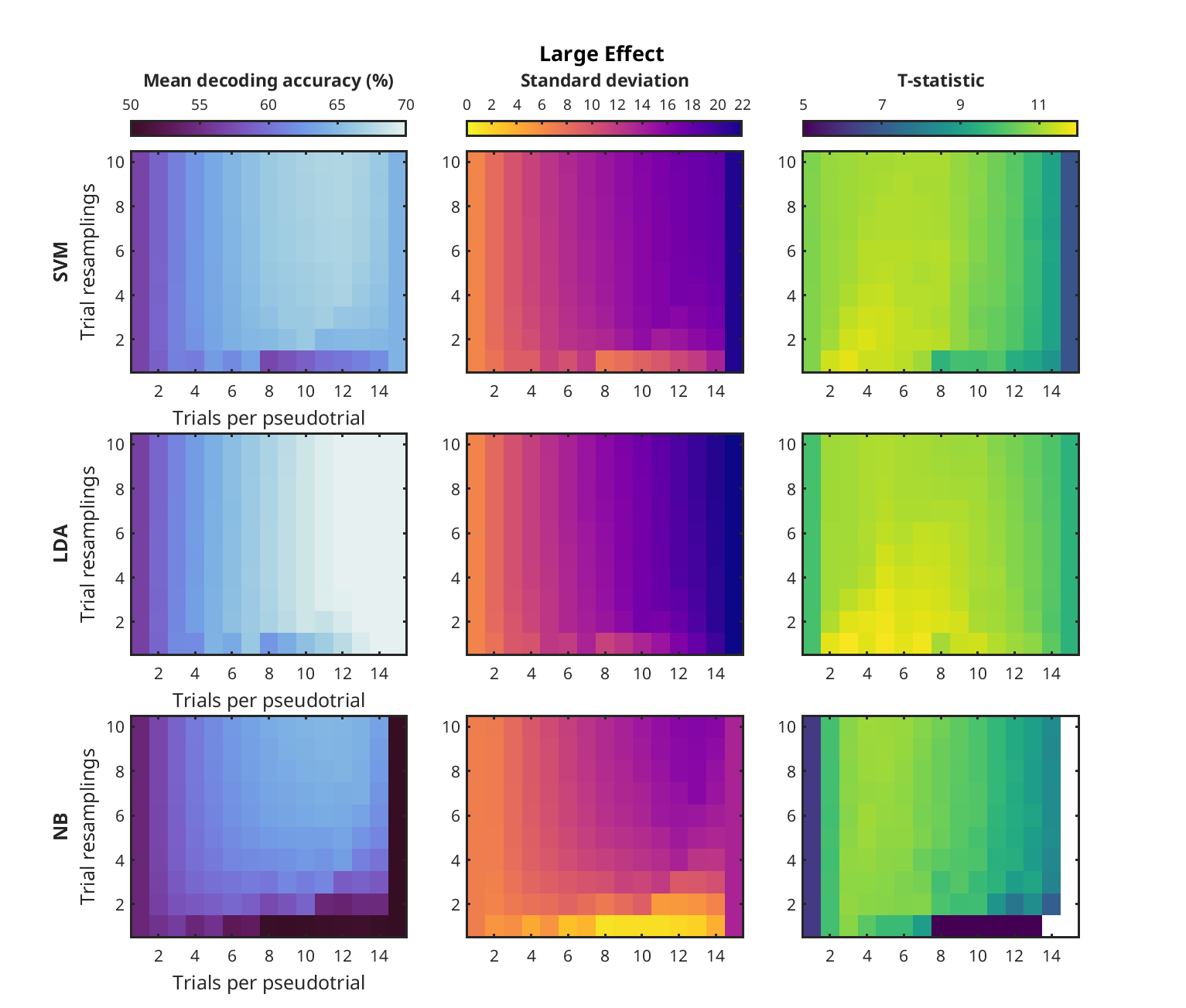
**

**Supplementary Figure 3.** The influence of both averaging and resampling for a dataset with only 45 trials per condition for simulated data with a large underlying effect. Results that were not significantly different from the permuted null using a sign-flip permutation test are masked out from the t-statistic plots (shown in white). Rows correspond to results from the three classifiers tested (SVM = support vector machine, LDA = linear discriminant analysis, NB = Naïve Bayes). Pseudotrials were created separately within 3 allocated ‘blocks’ of trials, facilitating a 3-fold cross-validation approach, with trials randomly allocated across 100 iterations of pseudotrial creation. For the results plotted here, we simulated data from 100 subjects with 45 trials per condition and large effect, or a class distance of 0.2 (see main Figure 4 for a small effect, or a class distance of 0.1).

# S3. Influence of a ‘leave-n-pseudotrials-out’ decoding approach (additional no effect and large effect)


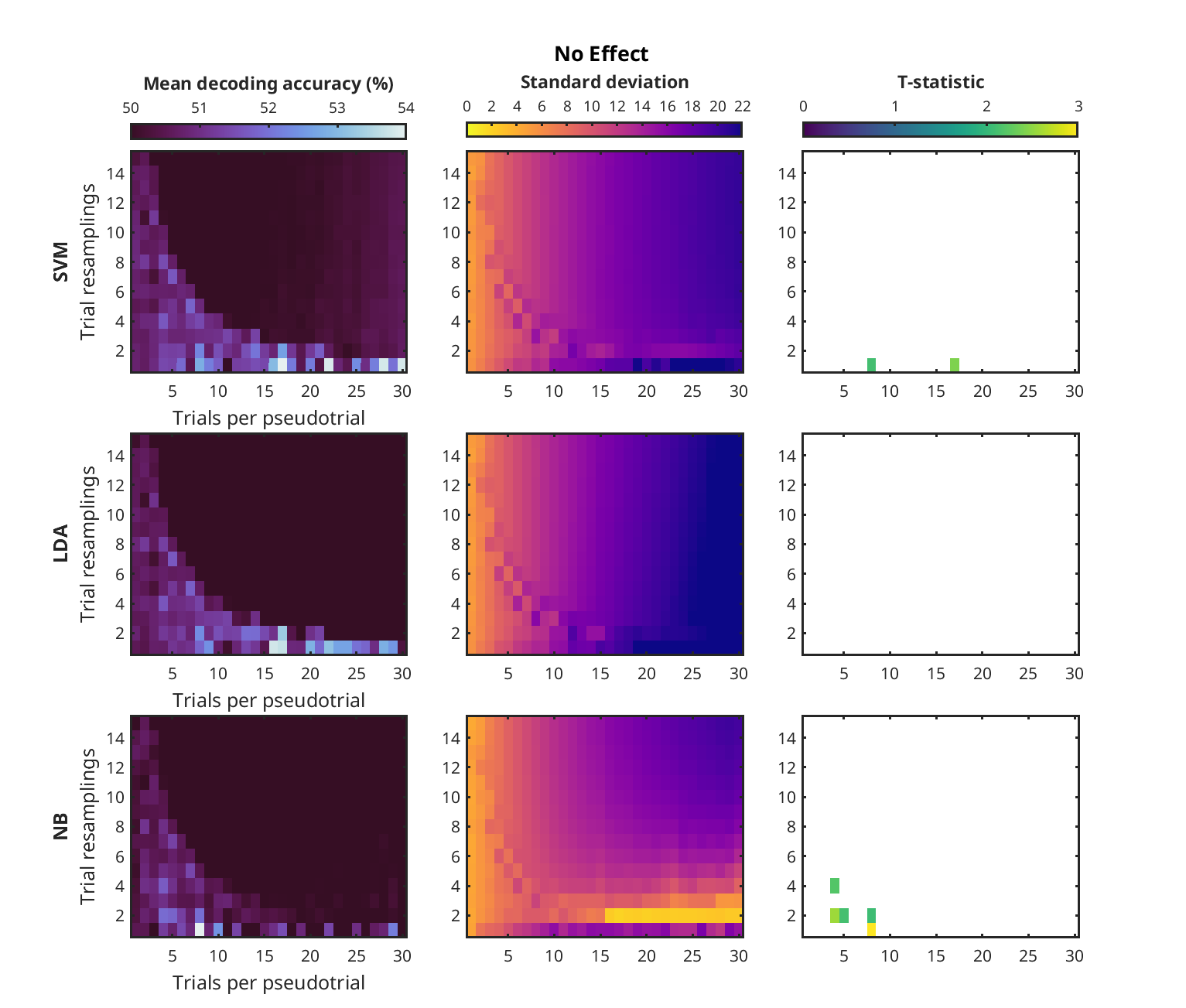
Here we examined a ‘leave-n-pseudotrials-out’ procedure, where the number of folds was determined by the number of pseudotrials created. First, we show the results with no simulated class distance. This differs from **main Figure 5** which displayed the results for a small effect (class distance of 0.1). Decoding performed on data with no simulated effect remained mostly at 50% (**supplementary Figure 4**). However, there were two false positive findings for the SVM classifier when using 8 or 17 trials per average with no resampling. This corresponds to decoding with 11 or 5 cross-validation folds. In addition, there were five false positive findings for the Naïve Bayes classifier when using between 4 and 8 trials per average, mostly at a resampling of 2. Note that we did not apply a correction for multiple comparisons as each decoding result reflects an independent simulated experiment rather than repeated tests on the same data; however, this may still increase the risk of false-positive findings. This very low number of false positives is within what we would expect to arise by chance when performing so many tests, rather than reflecting an effect of the pseudotrial method.

**Supplementary Figure 4.** The influence of averaging and resampling using a ‘leave-n-pseudotrials-out’ decoding approach for simulated data with no underlying effect. Results that were not significantly different from the permuted null using a sign-flip permutation test are masked out from the t-statistic plots (shown in white). Note that in the case of no underlying effect, we expect to find a non-significant decoding result for all tests, and coloured points indicate false positives. Rows correspond to results from the three classifiers tested (SVM = support vector machine, LDA = linear discriminant analysis, NB = Naïve Bayes). Here the number of cross-validation folds was determined by the number of trials per pseudotrial (the more trial averaging and resampling, the fewer possible folds), with trials randomly allocated across 100 iterations of pseudotrial creation. For the results plotted here, we simulated data from 100 subjects with 90 trials per condition and a large effect, or class distance 0 (see main Figure 5 for a small effect, or class distance of 0.1, and supplementary figure 5 for a large effect, or class distance of 0.2).

Next, we show the results for a ‘leave-n-pseudotrials-out’ procedure with a higher simulated class distance (0.2). This differs from **main Figure 5** which displayed the results for small effect (class distance of 0.1). For the Naïve Bayes classifier, high t-statistics were achieved when using roughly 15% of the original trials per pseudotrial (i.e., 4 or 5 trials) with a resampling of 2 (**supplementary Figure 5**). This is similar to the results with a small effect, or a class distance of 0.1 (**main Figure 5**). With a large effect (class distance of 0.2), the SVM classifier performed well with up to a third of the original trials (i.e., 10 trials) with a resampling of 2. For the LDA classifier, this could increase to half of the original trials (i.e., 15 trials) with a resampling of 2.


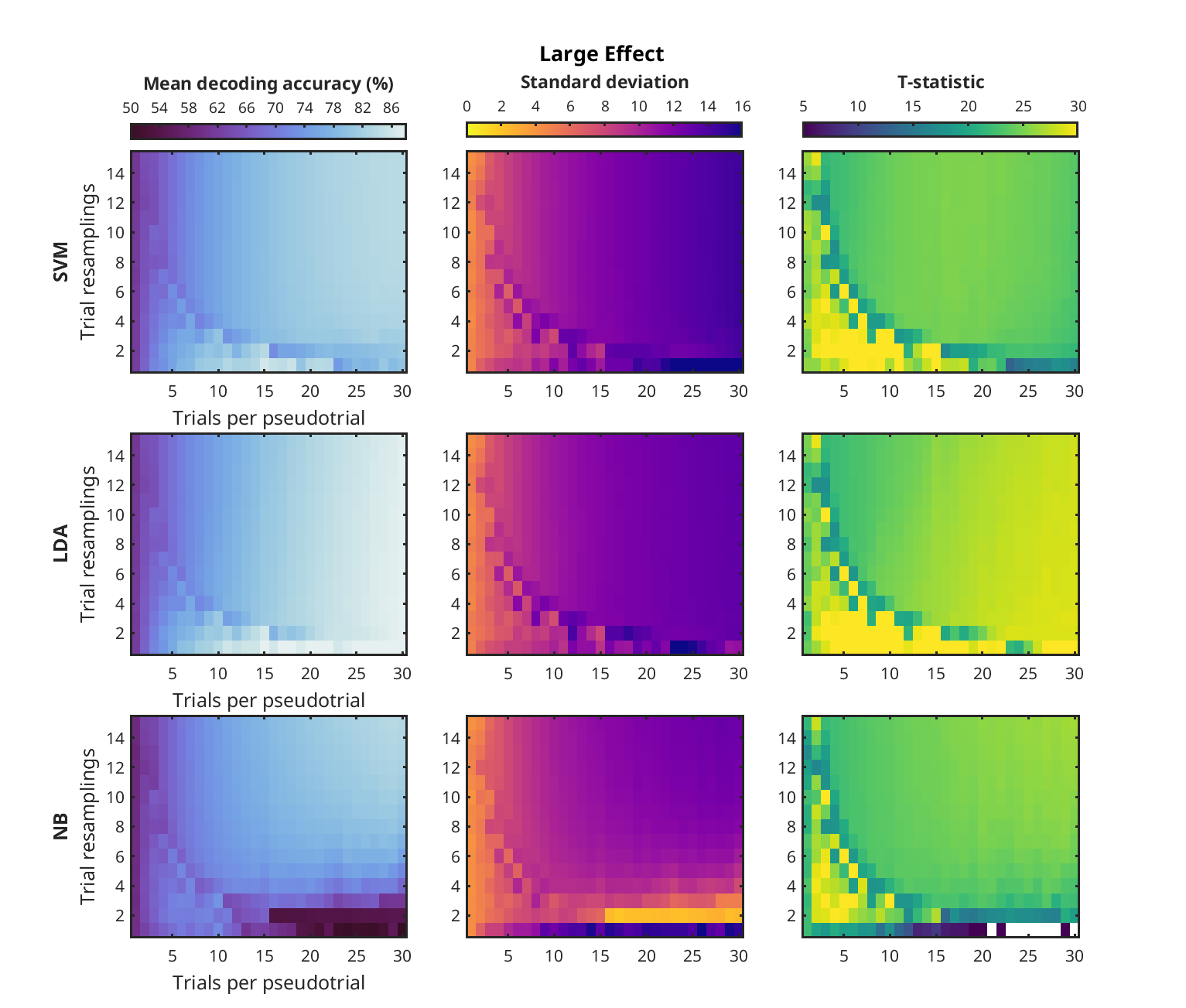


**Supplementary Figure 5.** The influence of averaging and resampling using a ‘leave-n-pseudotrials-out’ decoding approach for simulated data with a large underlying effect. Results that were not significantly different from the permuted null using a sign-flip permutation test are masked out from the t-statistic plots (shown in white). Rows correspond to results from the three classifiers tested (SVM = support vector machine, LDA = linear discriminant analysis, NB = Naïve Bayes). Here the number of cross-validation folds was determined by the number of trials per pseudotrial (the more trial averaging and resampling, the fewer possible folds), with trials randomly allocated across 100 iterations of pseudotrial creation. For the results plotted here, we simulated data from 100 subjects with 90 trials per condition and a large effect, or class distance of 0.2 (see main Figure 5 for a small effect, or class distance of 0.1, and supplementary figure 4 for no effect, or class distance of 0).

# S4. Influence of fewer ‘subjects’

Here we examined the influence of reducing the number of simulated ‘subjects’ from 100 to 50, which was not examined within the main text (**supplementary Figure 6**). A similar pattern is found to the data in **supplementary Figure 3** with 100 subjects and a large effect (a class distance of 0.2), although the overall performance is reduced. For the Naïve Bayes classifier, we found that using around a third of the original trials per chunk to create each pseudotrial (i.e., 10 trials) was sufficient to aid decoding. Combining this with a low resampling value of 2 further increased decoding accuracy. The SVM and LDA classifiers performed well with up to 50% of the original trials per pseudotrial (i.e., 15 trials) and a resampling value of 2.


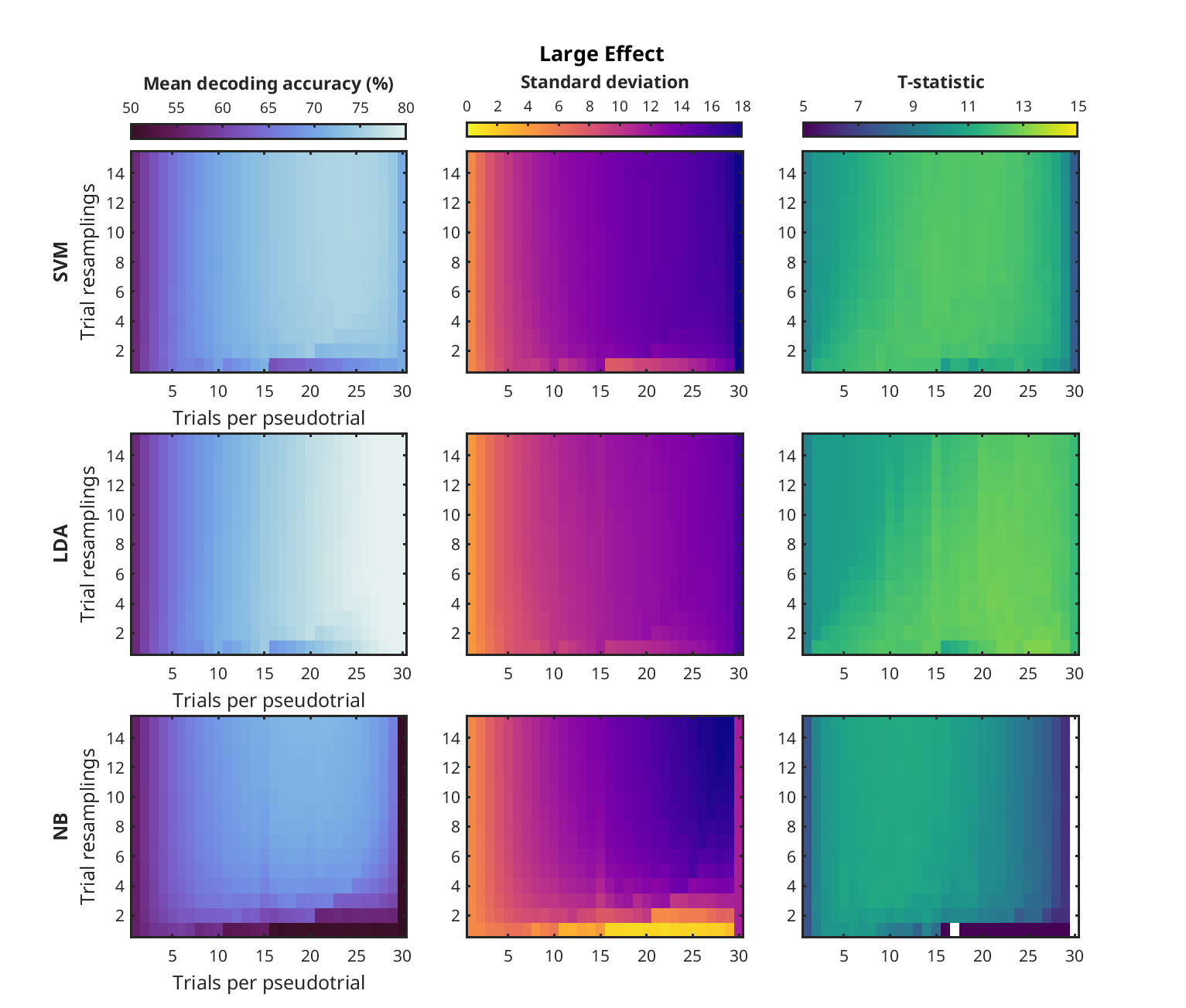


**Supplementary Figure 6.** The influence of both averaging and resampling with fewer subjects (n=50) for simulated data with a large underlying effect. Results that were not significantly different from the permuted null using a sign-flip permutation test are masked out from the t-statistic plots (shown in white). Rows correspond to results from the three classifiers tested (SVM = support vector machine, LDA = linear discriminant analysis, NB = Naïve Bayes). Pseudotrials were created separately within 3 allocated ‘blocks’ of trials, facilitating a 3-fold cross-validation approach, with trials randomly allocated across 100 iterations of pseudotrial creation. For the results plotted here, we simulated data from 50 subjects with 90 trials per condition and a large effect (class distance of 0.2).

# S5. Influence of a smaller number of features

Here we examined the influence of reducing the number of simulated features from 700 to 6, which was not reported in the main text. The overall performance of the classifiers was reduced, as well as the influence of the parameters on t-statistics. However, using around a third of the original trials per pseudotrial and a resampling of 2 would still be a reasonable choice to optimise classification performance.


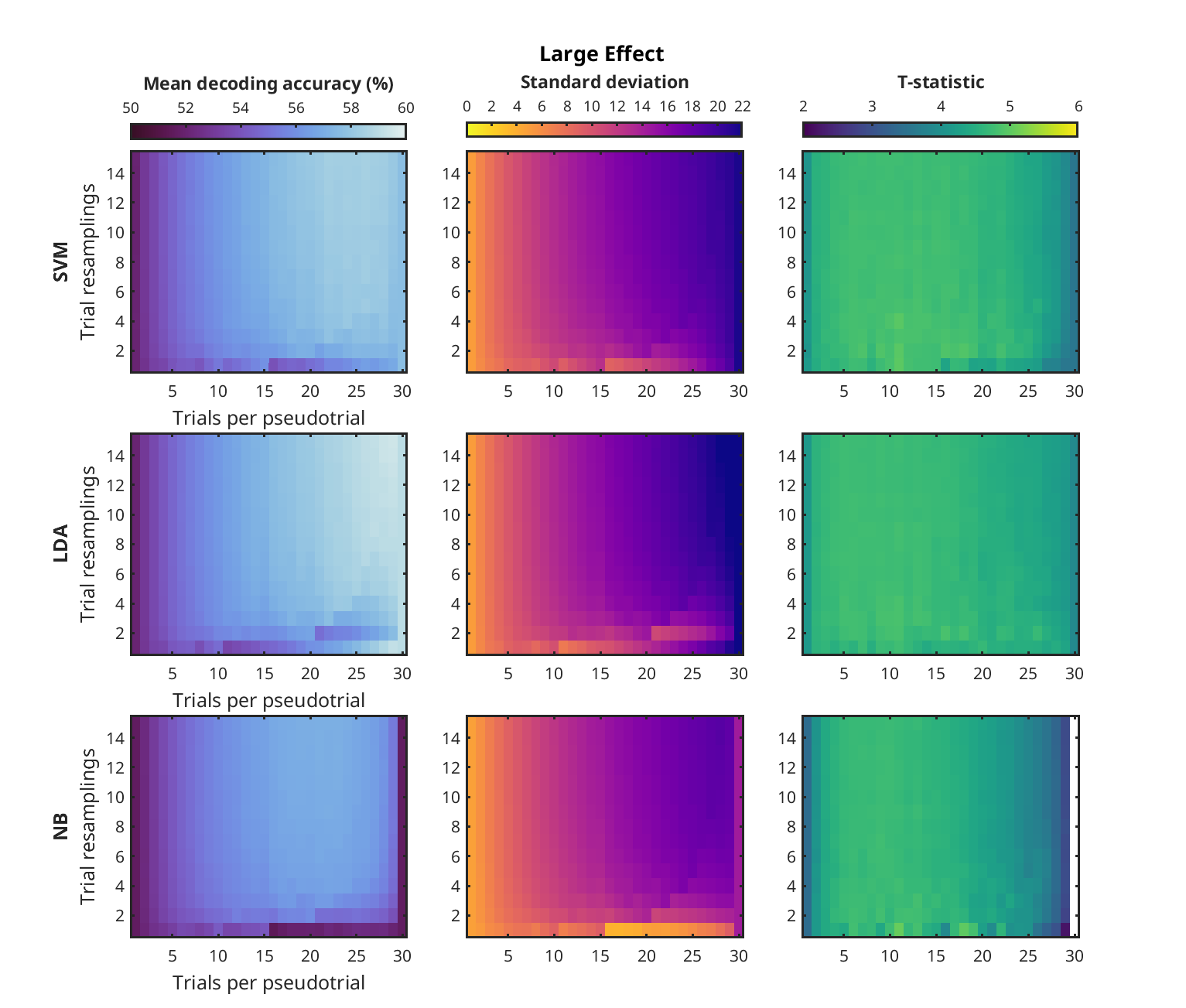


**Supplementary Figure 7.** The influence of both averaging and resampling with a smaller number of features (data size ‘small’ = 6 features) for simulated data with a large underlying effect. Results that were not significantly different from the permuted null using a sign-flip permutation test are masked out from the t-statistic plots (shown in white). Rows correspond to results from the three classifiers tested (SVM = support vector machine, LDA = linear discriminant analysis, NB = Naïve Bayes). Pseudotrials were created separately within 3 allocated ‘blocks’ of trials, facilitating a 3-fold cross-validation approach, with trials randomly allocated across 100 iterations of pseudotrial creation. For the results plotted here, we simulated data from 100 subjects with 90 trials per condition and a large effect (class distance of 0.2).

# S6. Influence of increasing the number of cross-validation folds (10-fold)

Here we examined the influence of increasing the number of cross-validation folds from 3 to 10. As we simulated 90 trials per condition, there was a maximum of 9 original trials per pseudotrial. As in the 3-chunk version (**main Figure 3**), using around a third of the original trials per chunk to create each pseudotrial (i.e., 3 trials) was sufficient to aid decoding. However, even for the Naïve Bayes classifier there was little to no benefit of resampling.

**
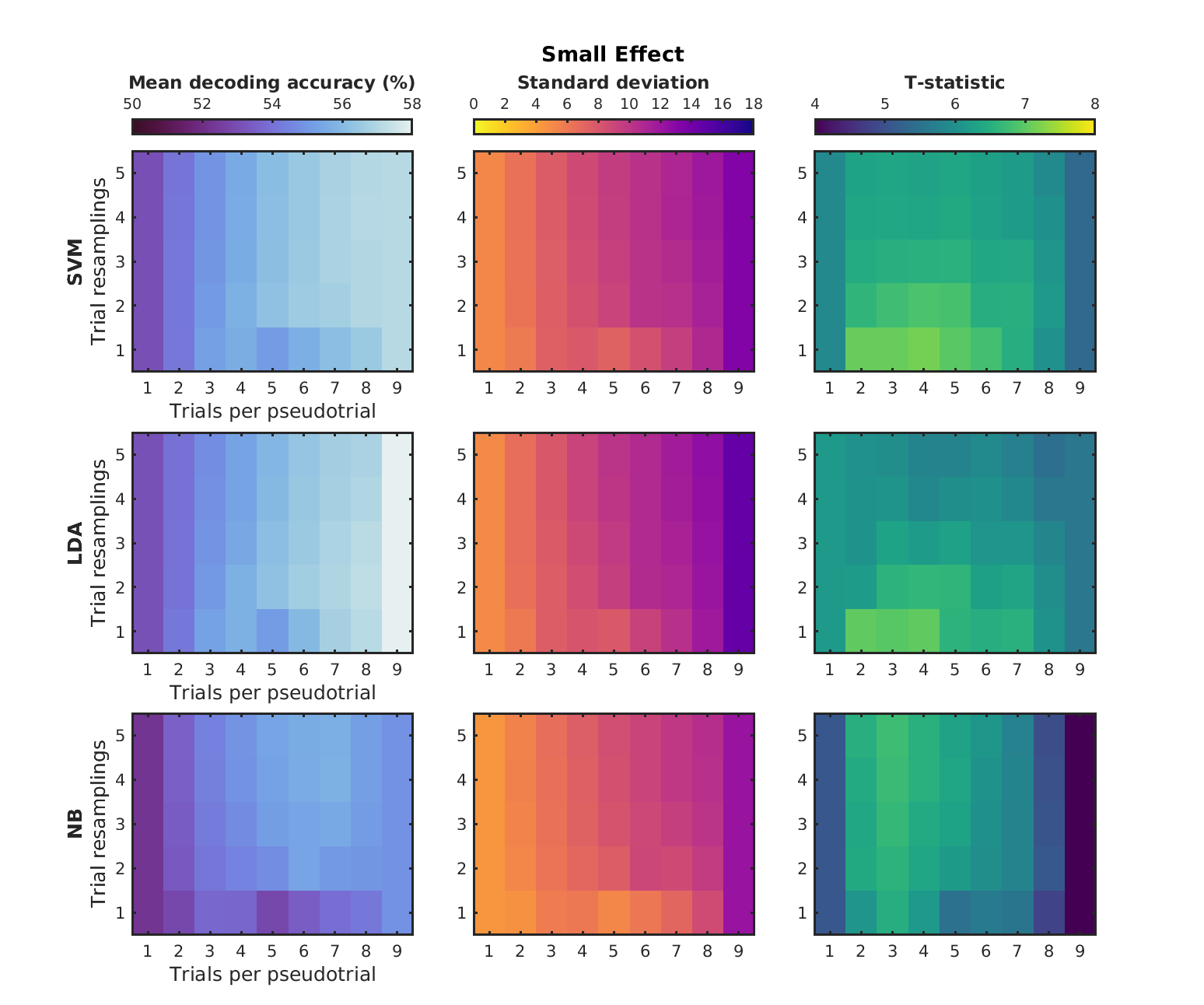
**

**Supplementary Figure 8.** The influence of averaging and resampling using a higher number of cross-validation folds for simulated data with a small underlying effect. Rows correspond to results from the three classifiers tested (SVM = support vector machine, LDA = linear discriminant analysis, NB = Naïve Bayes). Pseudotrials were created separately within 10 allocated ‘blocks’ of 9 trials each, facilitating a 10-fold cross-validation approach, with trials randomly allocated across 100 iterations of pseudotrial creation. For the results plotted here, we simulated data from 100 subjects with 90 trials per condition and a small effect (class distance of 0.1).

# **S7.** Influence of fewer iterations of random trial allocation

For the results reported in the main text, we ran 100 iterations of the pseudotrial procedure and averaged the 100 resulting classification accuracies. This was to ensure that the results were not dependent on the specific division of trails into pseudotrials, for each ‘subject’ and parameter set. **Supplementary Figure 9** demonstrates the pattern of results achieved with one, five, 50, and 100 iterations of random trial allocation. Without this iteration procedure (when the number of iterations = 1), trial averaging quickly increases the between-subject variance, meaning that little or no benefit is derived from the pseudotrial procedure. In fact, the pseudotrial procedure now becomes detrimental in most cases, especially for the NB classifier and for high numbers of trials/pseudotrial for SVM. Thus, including multiple iterations appears to be crucial for deriving benefit from the pseudotrial procedure.


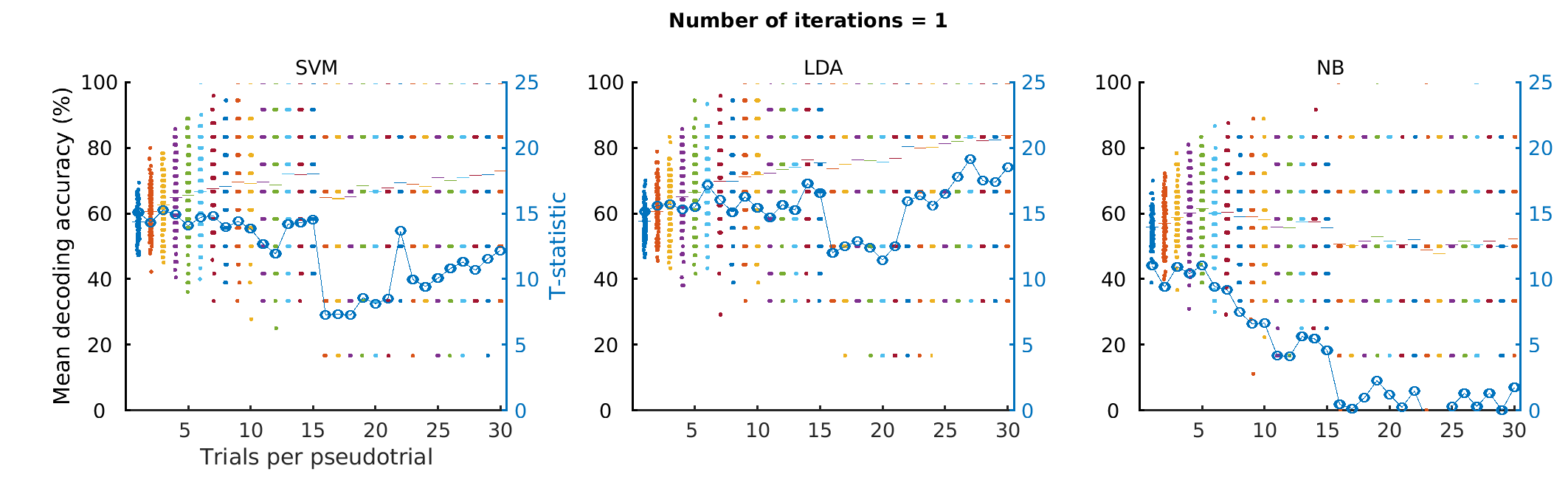

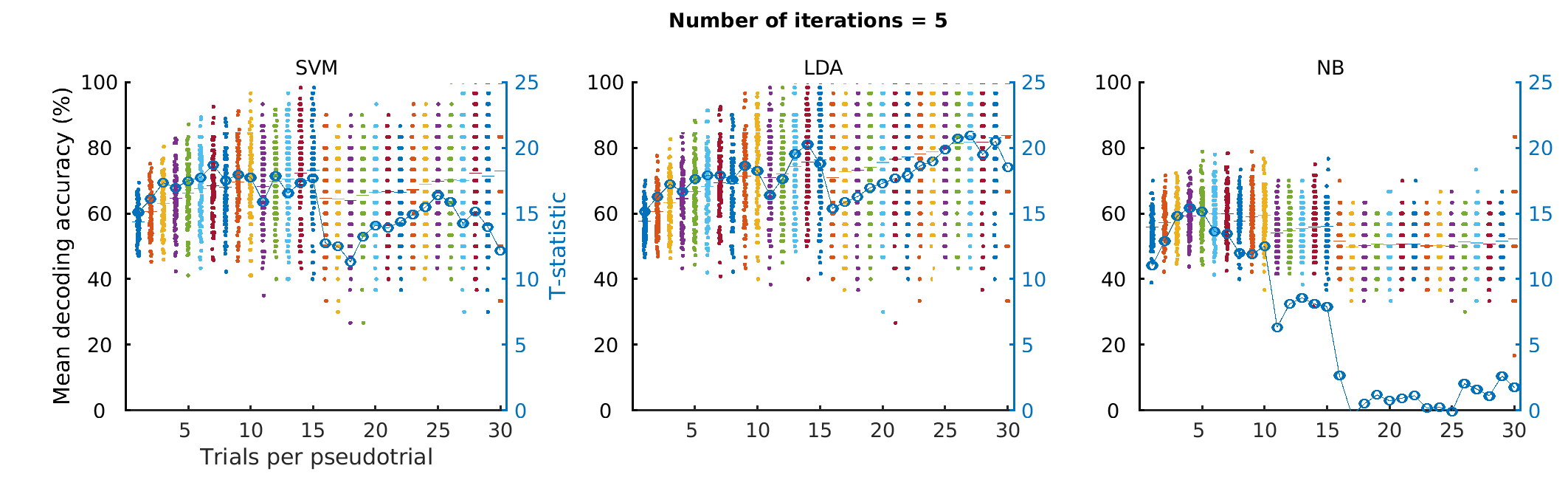

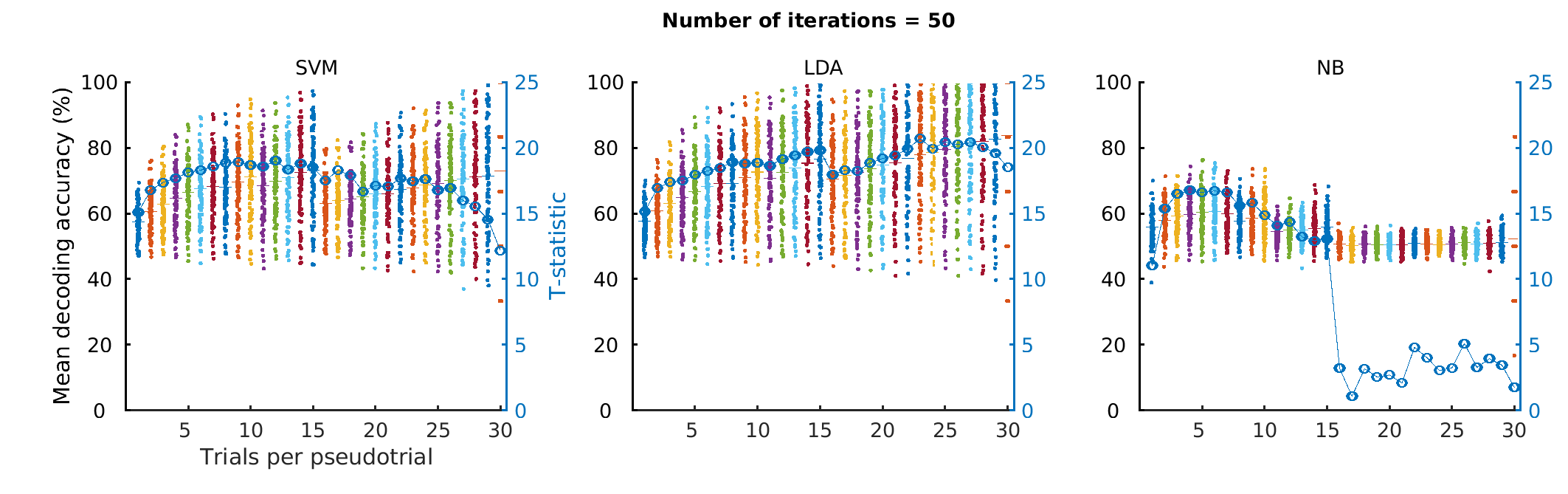

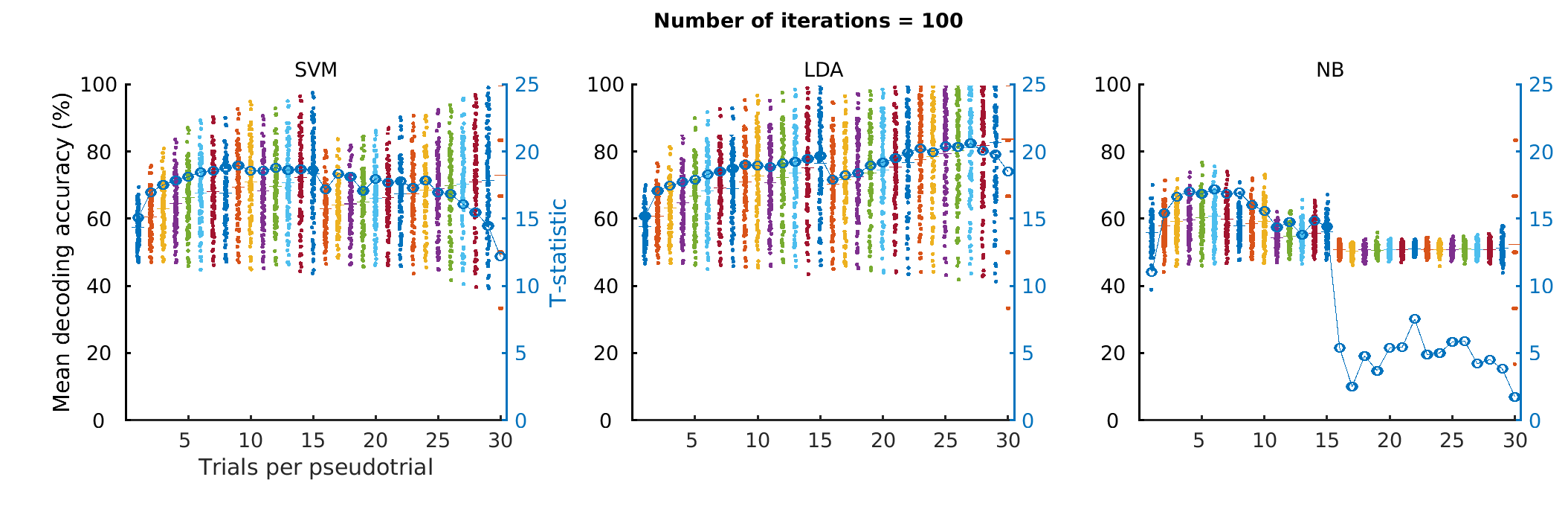


**Supplementary Figure 9.** The influence of fewer iterations of random trial allocation for simulated data with a large underlying effect. For the results plotted here, we simulated data from 100 subjects with 90 trials per condition and a large effect (class distance of 0.2). Rows correspond to the number of iterations of random trial allocation that was used to create pseudotrials. Columns correspond to results from the three classifiers tested (SVM = support vector machine, LDA = linear discriminant analysis, NB = Naïve Bayes). Pseudotrials were created separately within 3 allocated ‘blocks’ of trials, facilitating a 3-fold cross-validation approach, with trials randomly allocated.
